# Live fast, diversify non-adaptively: evolutionary diversification of exceptionally short-lived annual killifishes

**DOI:** 10.1186/s12862-019-1344-0

**Published:** 2019-01-09

**Authors:** Joshua W. Lambert, Martin Reichard, Daniel Pincheira-Donoso

**Affiliations:** 1 School of Life Sciences, Joseph Banks Laboratories, Brayford Campus, University of Lincoln, Lincoln, LN6 7DL UK; 2 The Czech Academy of Sciences, Institute of Vertebrate Biology, Brno, Czech Republic; 3 MacroBiodiversity Lab, Department of Biosciences, School of Science and Technology, Nottingham Trent University, Nottingham, NG11 8NS UK

**Keywords:** Macroevolution, Diversification, Non-adaptive radiation, Spatial opportunity, Speciation, *Nothobranchius*

## Abstract

**Background:**

Adaptive radiations are triggered by ecological opportunity – the access to novel niche domains with abundant available resources that facilitate the formation of new ecologically divergent species. Therefore, as new species saturate niche space, clades experience a diversity-dependent slowdown of diversification over time. At the other extreme of the radiation continuum, non-adaptively radiating lineages undergo diversification with minimal niche differentiation when ‘spatial opportunity’ (i.e. areas with suitable ‘ancestral’ ecological conditions) is available. Traditionally, most research has focused on adaptive radiations, while empirical studies on non-adaptive radiations remain lagging behind. A prolific clade of African fish with extremely short lifespan (*Nothobranchius* killifish), show the key evolutionary features of a candidate non-adaptive radiation – primarily allopatric species with minimal niche and phenotypic divergence. Here, we test the hypothesis that *Nothobranchius* killifish have non-adaptively diversified. We employ phylogenetic modelling to investigate the tempo and mode of macroevolutionary diversification of these organisms.

**Results:**

*Nothobranchius* diversification has proceeded with minor niche differentiation and minimal morphological disparity among allopatric species. Additionally, we failed to identify evidence for a role of body size or biogeography in influencing diversification rates. Diversification has been homogeneous within this genus, with the only hotspot of species-richness not resulting from rapid diversification. However, species in sympatry show higher disparity, which may have been caused by character displacement among coexisting species.

**Conclusions:**

*Nothobranchius* killifish have proliferated following the tempo and mode of a non-adaptive radiation. Our study confirms that this exceptionally short-lived group have diversified with minimal divergent niche adaptation, while one group of coexisting species seems to have facilitated spatial overlap among these taxa via the evolution of ecological character displacement.

**Electronic supplementary material:**

The online version of this article (10.1186/s12862-019-1344-0) contains supplementary material, which is available to authorized users.

## Background

Ecological opportunity – the instance whereby novel environments with abundant resources become available – is a widespread source of divergent natural selection that can trigger the proliferation of an ancestor into multiple species adapted to different regions of the niche space [1–4]. Therefore, lineage diversification via this process, known as adaptive radiation [2], is expected to leave a phylogenetic signature consisting of an ‘early burst’ of species accumulation via niche-filling, followed by a slowdown in diversification rate as newly emerging species saturate ecological opportunity [5–9]. Consequently, adaptive diversification is diversity-dependent, as saturation of niche space reaches the ecological capacity of a given environment [10–12].

Evolutionary radiations are not exclusively adaptive [13], however, and can take place when species diversify with retention of the ancestral niche [14]. During this process of non-adaptive radiation [15], episodes of speciation are not the result of divergent natural selection and thus, newly emerging species remain ecologically and phenotypically similar [16]. Importantly, it has been suggested that in contrast to adaptive radiations, diversification via non-adaptive radiation is more likely to take place when newly emerging species radiate across geographically non-overlapping areas (allopatry), which can accommodate species with fundamentally similar niche demands [15, 17, 18]. The availability of such areas equipped with suitable ‘ancestral’ ecological conditions is what we refer to as ‘spatial opportunity’. Given that this form of radiation is bound by the availability of spatial opportunity, rather than by environments with an abundance of diverse niche space, the process of species accumulation is not expected to always exhibit a slow-down over time. Instead, an arithmetic accumulation of species is expected when incipient species encounter new spatial opportunity to continue clade expansion producing a phylogenetic late-burst [18]. Alternatively, if an abundance of spatial opportunity is available and exploited early in the clades’ history, it will produce an early burst, akin to an adaptive radiation [8]. Consequently, depending on the availability of vacant ancestral niches, non-adaptive radiations may diversify under a range of radiation trajectories.

Empirical investigations quantifying the tempo and mode of adaptive diversification have been historically popular and conducted across an array of continental and island radiations [6, 19–23]. The fundamental principles underlying non-adaptive radiations are well-studied ([24] and references therein). In contrast, the phylogenetic characterisation of the tempo and mode of non-adaptive radiations has emerged more recently and remains a pending task in our understanding of the role of adaptation during diversification [8, 18, 25, 26]. The extent to which niche and phenotypic disparity are preserved across prolific clades, and the impact of coexistence among some of the species to exert a triggering phenomenon such as ecological character displacement remain poorly known. Body size is frequently used to investigate both phenotypic and niche evolution across adaptive and non-adaptive radiations due to its central role in ecological and evolutionary processes and evolutionary correlation to other character traits [27–30].

**Fig. 1:**
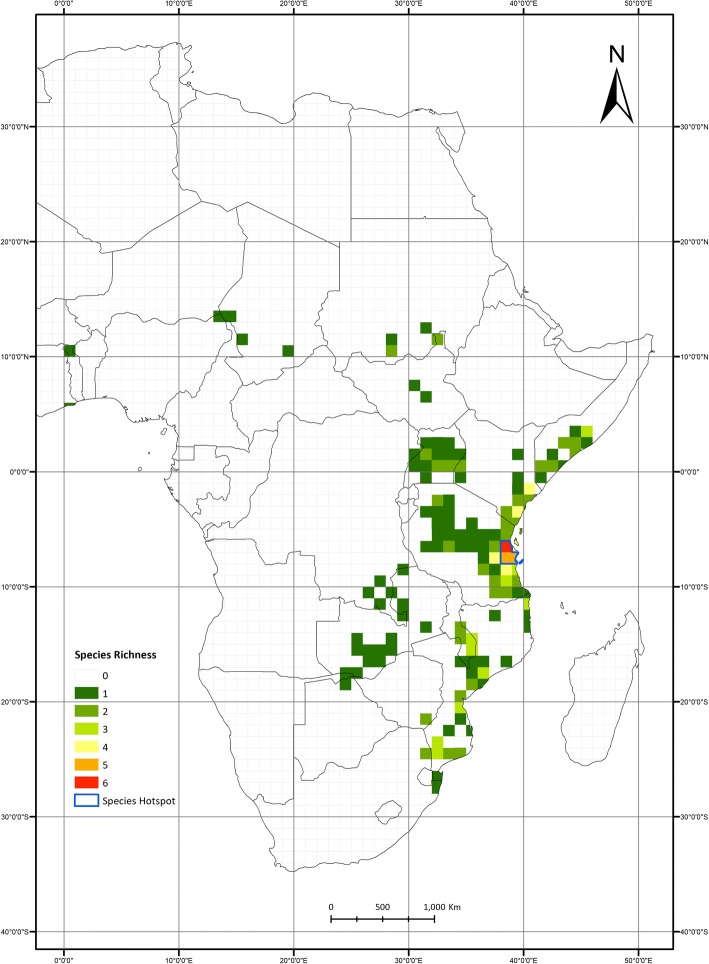
*Nothobranchius* species richness map. Richness and distance scale bottom left, with the blue outline representing the species hotspot. The mapped distributions are based on original data and the map was created using ArcGIS v10.0

The *Nothobranchius* genus of African killifish consists of over 70 freshwater species adapted to annually desiccating savannah pools, and which survive desiccation by diapausing embryos during the dry season specifically in alluvial vertisol soil [31–34]. As a result, *Nothobranchius* species have evolved extremely short lifespans, often limited to a few weeks [35, 36]. The genus shows a history of vicariance-driven diversification, with the mode of dispersal between pools still unclear [37, 38]. *Nothobranchius* species (71 [39]) have predominantly radiated allopatrically, currently ranging from Sudan to South Africa along the East of Africa, including Zanzibar and Mafia Islands [37, 40]. This spatial structure is coupled with minimal ecological and morphological disparity among species [40–42]. An area of lowland Tanzania apparently deviates from this tendency, as a high number of species coexist over a relatively small area, forming the only hotspot of *Nothobranchius* species richness [37, 43]. This hotspot (Fig. 1) is hypothesised to have resulted from dispersal into the hotspot or high diversification, either via secondary contact after speciation in allopatry, or via sympatric speciation. Both hypotheses predict that the interspecific coexistence within this area is conditioned by the evolution of ecological character displacement among species, which could have taken place during sympatric speciation (i.e. consistent with a process of adaptive diversification) or from divergent selection pressures forcing species to diversify phenotypically during earlier stages of allopatric diversification prior to secondary contact.

Here, we employ phylogenetic modelling to investigate the tempo and mode of macroevolutionary diversification of the genus *Nothobranchius*. These analyses are implemented to test the core hypothesis that the *Nothobranchius* clade has predominantly radiated via non-adaptive diversification. The tempo of diversification was investigated by determining whether diversification was heterogeneous through time and across the phylogeny. Non-adaptive diversification was tested by modelling the evolution of body size to elucidate the evolutionary process that produced the pattern of minimal phenotypic disparity. We investigate whether any diversification heterogeneity across the phylogenetic tree is the result of species’ body size, biogeography, or an unmeasured trait. Finally, by investigating the effect of biogeography on diversification we explore why a hotspot has arisen and whether these species have shifted along the ‘radiation continuum’.

## Methods

### Data collection

To quantify diversification in phenotype and the role of the phenotype in diversification rates, we gathered a dataset of body size for 48 *Nothobranchius* species, using maximum standard length (SL_max_) (a linear measure from the anterior tip down to the midlateral posterior of the hypural plate). Body size represents the primary descriptor for niche space in fishes as it integrates the most significant information on ecological characteristics of particular species [28, 44]. Measures of body size were obtained from the data collected directly by one of us (MR), and collected from multiple literature sources (Additional file 1). Prior to all analyses, body size data were transformed into natural logarithm to meet requirements of data distribution of variance homogeneity (Additional file 2). Secondly, we created a new dataset consisting of all occurrence records known for all *Nothobranchius* species present in the phylogenetic tree (see below). These data were obtained from extensive field records collected over the course of 10 years by one of us (MR, [45]), as well as from coordinates recorded by overlapping the maps published by Wildekamp [46] with a geographic information system and a set of secondary resources (Fig. 1; Additional files 3 and 4).

### Phylogenetic tree

Macroevolutionary phylogenetic analyses of diversification were performed on a time-calibrated molecular phylogenetic tree that includes 49 of the 71 documented *Nothobranchius* species from Dorn et al. [37] (Additional file 5; [39]). The tree was built based on the analysis of five nuclear markers and one mitochondrial marker; fossil-based calibration was not possible due to the lack of a *Nothobranchius* fossil record, but used secondary calibration from a fossil-calibrated tree of spiny-rayed fish [37, 47]. For lineage diversification analyses and biogeographic analysis we used the full phylogeny (*n* = 49). For body size and trait-dependent diversification analyses, we used a phylogenetic tree of the 48 species for which these data were available.

### Lineage diversification analyses

Tempo of diversification – the relationship between speciation and extinction rate – was qualitatively analysed using a lineage-through-time (LTT) plot using the package ‘ape’ [48] in R version 3.3.1 [49]. The LTT is plotted against a pure-birth null model displayed as confidence intervals ranging from 50 to 99%, to identify significant pulses of lineage accumulation. Taxa missing from the incomplete phylogeny (*n* = 49) are not corrected for in LTT analysis. The gamma (γ) statistic was calculated using phylogenetic node distances to detect whether the tempo of diversification is significantly different from the diversification tempo of a pure-birth null model, and can be used to quantify whether diversification underwent an early burst or late burst [50]. A property of the ultrametric tree is all tips (i.e. extant taxa) are of equal distance to the root of the tree (i.e. common ancestor), thus the bifurcation events that occur indicate tempo changes through time [51]. The γ statistic was produced using the R package ‘ape’ [48], to test for significance a critical value was calculated by running a Monte Carlo Constant Rate (MCCR) analysis using R package ‘laser’ [52]. MCCR analysis accounts for species absent from the phylogenetic tree, as an increase in type I error and negative bias arises when using an incomplete phylogeny [53]. Bias-correction used a proposed clade size of 71 *Nothobranchius* species. MCCR analysis was run for 10,000 iterations.

Hidden Markov model based maximum-likelihood analysis of clade diversification was conducted using R package ‘DDD’ [8]. Four models derived from the birth-death process were fitted to analyse whether diversification rate is constant or a function of species richness. Two diversity-independent models were fitted: the Yule model (pure-birth model), defined by a non-zero speciation rate and zero extinction rate, and a constant rate birth-death (crBD) model with non-zero speciation and extinction rates. In addition, two diversity-dependent models were fitted: diversity-dependent linear (DDL + E), and diversity-dependent exponential (DDE + E) models, that model speciation rate as a linear and exponential function of number of species in the phylogeny at any point in time, respectively, while accounting for a non-zero extinction rate [8]. Goodness-of-fit was established by ranking model on the bias-corrected Akaike Information Criterion (AICc) using maximum likelihood values showing the best-fitting model with minimum information loss [54, 55]. Similar to the MCCR test, clade diversification analysis incorporates taxa absent from the phylogeny. In the case of *Nothobranchius*, the phylogenetic tree assemblage of 49 is missing 22 species from the proposed complete clade of 71 [39]. All models were re-run to check whether model selection is consistent when the proportion of missing species changes, to account for the number of divergent populations that may be potentially described as separate species, as the conservative number of described species may bias diversification towards diversity-dependence. Parametric bootstrap likelihood ratio tests – at a significance value (α) of 0.05 – were used to ensure the best-fitting models chosen using AICc were reliable [56]. The best-fitting diversity-dependent model (DDL + E or DDE + E) from maximum likelihood analysis was compared with the constant rate birth-death model. The likelihood ratio test was carried out for 1000 bootstrap iteration at both 22 and 50 missing taxa.

To detect whether diversification rate is heterogeneous through time, we analysed the phylogenetic tree for rate shifts using Bayesian Analysis of Macroevolutionary Mixtures (BAMM) version 2.5 [57]. Phylogeny-specific priors for the number of diversification shifts, speciation and extinction parameters were estimated using the ‘BAMMtools’ package in R [58]. The priors are estimated based on the root age of the phylogenetic tree and using a pure-birth model. These estimates are subsequently used by BAMM to calculate a lineage-specific posterior distribution of speciation and extinction rates, which are then used to model diversification shifts. BAMM uses a reversible jump Markov Chain Monte Carlo (MCMC) approach, which we ran for 10,000,000 iterations to ensure convergence on four metropolis-coupled MCMC chains (deltaT = 0.1, swapPeriod = 1000), to detect diversification rate-shifts. BAMM incorporates species missing from the phylogeny, by assuming 22 species are missing and that phylogenetic species are sampled at random [59]. The R package ‘coda’ [60] was used to ensure MCMC convergence (effective sample size [ESS] for likelihood and number of shifts > 500) for posterior distribution accuracy after a 10% burnin. Model goodness-of-fit was delineated using Bayes factors (BF) from BAMM analysis [61], for a non-zero shift model to be accepted over the null hypothesis it must have a BF over 3 [62].

### Phenotypic diversification analyses

Diversification of phenotypic traits was examined by trait disparity-through-time (DTT) analysis using the R package ‘geiger’ [63, 64]. This analysis uses body size of extant *Nothobranchius* species to reconstruct ancestral body size values and model disparity between species. DTT is carried out by the average squared Euclidean distance between species on a Euclidean (multivariant) space [19]. The null model of trait evolution is Brownian motion (BM), a stochastic evolution model of constant variance, produced by averaging 10,000 BM iterations [65]. Confidence intervals for DTT were calculated using the rank envelope test as this shows better type I and II error rate than the original pointwise envelope test [66]. Morphological disparity index (MDI) quantified the disparity from the reconstructed evolution of body size from extant species and the median trait values under Brownian evolution simulations. Whereby a positive MDI represents a greater disparity than Brownian expectation, conversely a negative MDI represents less disparity than Brownian expectation [19, 67]. Ancestral state reconstruction of body size was carried out using a maximum likelihood BM model of trait evolution and plotted onto the phylogenetic tree using the R package ‘phytools’ [65, 68, 69].

The quantification of body size evolution was carried out by fitting four models of trait evolution: Brownian motion (BM), Ornstein-Uhlenbeck (OU), Early-Burst (EB), and the Delta model using the R package ‘geiger’ [21, 63, 64]. BM models trait evolution between species as a correlation to the time since divergence as the trait values evolve under a random pattern with variance determined by the scaling parameter (σ) [65]. OU models trait evolution under Brownian motion with an additional stabilizing selection term, driving towards an optimum value (θ) at a rate of adaptation (α) [70, 71]. The EB model of evolution is the exponential decrease in trait evolution over time, indicative of the early divergence in adaptive radiations [21]. The Delta model of evolution detects the changes in tempo of trait evolution [72]. The delta (δ) statistic infers whether trait evolution happens early in the clades’ history and then experienced a slow-down (δ < 1), analogous to EB; or whether the trait evolution has occurred in the relative recent past (δ > 1), suggestive of adaptation to new fitness optima. When δ = 1 it indicates constant tempo through the lineage, equal to the tempo described by the BM model. All models assume gradual trait evolution, not testing for punctuated equilibrium [73]. Goodness-of-fit was determined using AICc.

We conducted an a posteriori approach to determine whether body size evolution is under a single- or multiple-optima OU model; testing whether morphology has converged around one or more fitness optima. This was carried out using the R package ‘SURFACE’ [74] and ‘ℓ1ou’ [75]. SURFACE determines the number and location of selection regimes (OU optima) across the phylogeny. Successive regimes are modelled across the tree using a stepwise approach until best-fit model using AICc, then regimes are collapsed until the most parsimonious regimes and shifts are chosen using AICc [74]. This stepwise AICc method has limitations in its false positive detections rate for optima shifts [75, 76]. The ℓ1ou uses a phylogenetic lasso method to detect and locate OU shifts, resolving to some extent the limitations of the SURFACE method by using a phylogenetic Bayesian information criterion (pBIC) [75]. To quantify support for each shift detected using ℓ1ou, bootstrapping was run for 1000 iterations. These methods are used comparatively as SURFACE will detect small magnitude OU shifts in a few or single species, while ℓ1ou will provide a more conservative estimate of shifts. Adaptive peaks were plotted onto a phenogram of reconstructed ancestral lineage’s body size using maximum likelihood BM in the R package ‘phytools’ [65, 68, 69].

To determine whether *Nothobranchius* body size evolution displayed significant rate shifts throughout the phylogeny and whether this would mirror any diversification shifts we ran BAMM for trait evolution [57]. Priors for rate of trait evolution and expected number of shifts were calculated using the ‘BAMMtools’ R package [58]. The trait priors are estimated based on the root age of the phylogenetic tree and maximum likelihood under a Brownian motion model. BAMM calculates posterior distributions to model trait evolution. BAMM was implemented under the same procedure as diversification analysis (see above). MCMC convergence (likelihood and number of shifts ESS > 500, burnin 10%) was tested using the R package ‘coda’ [60]. BF were calculated for model selection [61].

### Modelling trait-dependent diversification

In order to determine whether the rate at which lineages diversify is dependent on that lineage’s body size we ran trait-dependent diversification analysis using; a tip rate correlation technique termed inverse of equal-splits with simulated null model (*ES-sim*) [77], and maximum likelihood approach Quantitative State Speciation Speciation and Extinction (QuaSSE) [78]. These were used comparatively as for a phylogeny of ~ 50 species *ES-sim* provides a better Type I error rate but QuaSSE has higher statistical power [77]. ES*-sim* measures the tip-specific speciation rate and simulates a null distribution under a given model of trait evolution in order to test for significance. We ran *ES-sim* for both Brownian and OU null distributions by running 10,000 simulations, using the ‘essim’ code in R [77]. Spearman’s correlation was used to determine a significant monotonic (i.e. linear and sigmoidal) trait-dependent diversification relationship using body size data of extant species. QuaSSE is a state-dependent speciation-extinction model based on BiSSE [79] using the R package ‘diversitree’ [80]. Analysis using a birth-death process was performed using the phylogenetic tree and body size data to model: linear-, sigmoidal- and modal-diversification, for both stochastic trait evolution, and directional trait evolution [78]. Assuming random sampling, missing species are corrected for to avoid speciation-extinction bias for an incomplete phylogeny [59]. QuaSSE analysis calculates maximum likelihood for model selection, from which AIC was calculated to delineate model goodness-of-fit.

### Biogeographic analysis

Of particular interest is a region of high species density in Tanzania. The boundary of the hotspot is delineated using 1^o^ latitude × 1^o^ longitude grid cells and includes all cells with greater than 10% of *Nothobranchius* species in the phylogeny (Fig. 1). To test whether species’ biogeography influences diversification we tested for biogeographic-dependent diversification using a geographic state and hidden state speciation-extinction model (GeoHiSSE) analysis, using R package ‘HiSSE’ [81–83]. This model uses a three-state Markov Chain, to allow for species that live in distinct biogeographical area (A or B), or occupy both areas (AB). Parameters of the model are speciation rate in each area (sA, sB) as well as both areas (sAB); the extinction rate of each area (xA, xB), and dispersal between areas (dA = A➔B, dB = B➔A). Missing species are corrected for using the same technique as QuaSSE [59]. We fit a seven parameter model without hidden states (GeoSSE), an equal model with hidden states (GeoHiSSE), and then two character-independent null models with hidden states (CID GeoSSE and CID GeoHiSSE). The null models have equal complexity to ensure for proper hypothesis testing and that the importance of geography in the formation of the hotspot [83]. Best-fit models were chosen using AICc, while parameter estimates are taken from model-averaging using Akaike weights (AICw). We do not attempt to delineate between range contraction and widespread species extinction in one geographic region due to insufficient phylogeny size.

## Results

### Tempo and mode of lineage diversification

**Fig. 2:**
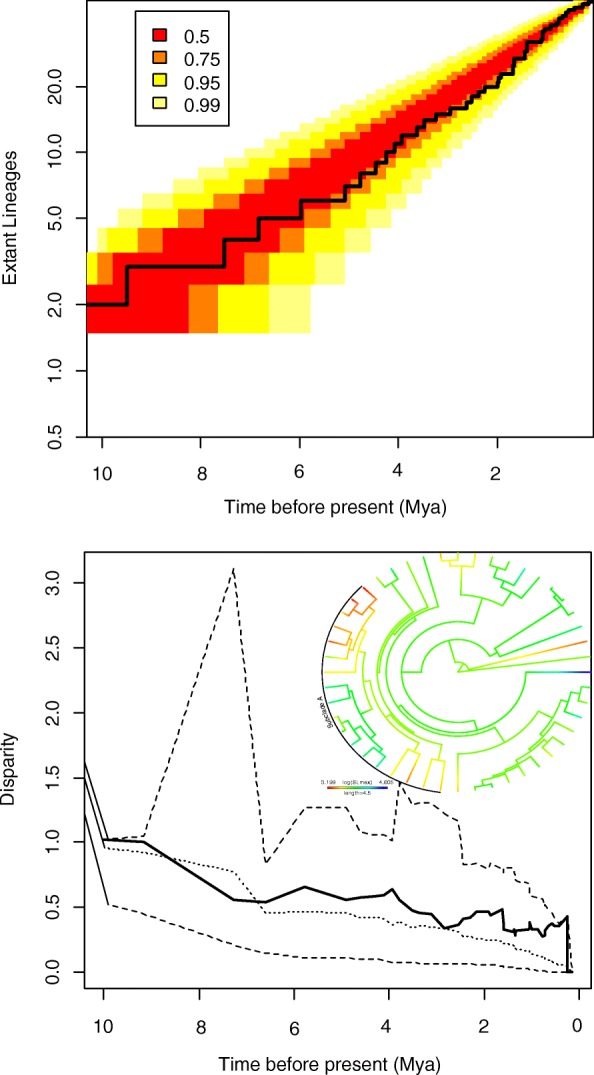
Top, lineage-through-time (LTT) accumulation curve for *Nothobranchius* radiation (black line). Unit of time (x-axis) is in millions of years before present (Mya). Coloured area represents confidence intervals for pure-birth model. Confidence intervals range from 50% (0.5) to 99% (0.99). Bottom, disparity-through-time (DTT) for body size (solid line), against a Brownian motion null model (dotted line). Area within dashed lines is the confidence intervals at 95%. Time before present in in millions of years before present. Inset in the DTT plot is the Brownian motion reconstruction of body size across each lineage with subclade A labelled

The accumulation of lineages is constant through time. Diversification is consistent with the pure-birth null model through the clades’ phylogenetic history (Fig. 2). A non-significant pulse in the late Pleiocene-early Pleistocene (~ 2.5–2 Mya), possibly represents a decreased rate of diversification. The slightly negative γ value (γ = − 0.145) indicates that the branching events of the reconstructed phylogenetic tree possesses a higher density of internal nodes marginally closer to the root of the tree than the tree tip compared to the pure-birth null model. The MCCR analysis found a critical value to reject a pure-birth model of − 2.298, consequently the γ statistic is highly non-significant (*p* = 0.731, 95% CI one-tailed test), rejecting an early- or late-burst of diversification.

Lineage diversification analysis elucidated that the Yule model was the best-fit for a proposed clade size of 71 and 99 species (Table 1). Constant rate birth-death also explained the data closely, while diversity-dependent models showed markedly less support (ΔAICc> 2), suggesting lineage tempo has not decreased with time. Comparison of the likelihood ratio tests from parametric bootstrapping was in accordance with the maximum likelihood analysis by rejecting the diversity-dependent (DDL + E) in favour of the constant rate birth-death model for both clade sizes (Additional file 6a, b, Additional file 7a and b). Therefore, all diversification analyses are congruent indicating no significant slow-down in diversification through time.

**Table 1:** Diversity-dependent diversification maximum likelihood analysis using bias-corrected Akaike Information Criterion (AICc) for model selection

Model	Taxa absent	λ	μ	LogL	AICc	ΔAICc
Yule	22	4.47	0	13.72	−25.35	0
crBD	22	5.28	1.53	13.96	−23.66	1.69
DDL + E	22	6.63	2.56	14.03	−21.53	3.82
DDE + E	22	6.29	2.26	13.90	−21.27	4.08
Yule	50	5.24	0	12.63	−23.18	0
crBD	50	7.37	3.62	13.66	−23.06	0.12
DDL + E	50	8.39	4.33	13.68	−20.82	2.38
DDE + E	50	8.19	3.40	13.61	−20.69	2.49

Yule (pure-birth), constant rate Birth-Death (crBD), diversity-dependent linear speciation + extinction (DDL + E), and diversity-dependent exponential speciation + extinction (DDE + E) models were run. Maximum likelihood parameter estimates of speciation rate (λ) and extinction rate (μ) from each model. All models were run on two sets of missing taxa, 22 and 50

BAMM analysis corroborates the constant rate through time with constant rate across lineages. After ensuring the MCMC had converged (likelihood ESS = 1461.15, number of shifts ESS = 1398.48), model comparison using BF showed that the null model with no shifts in diversification rate was the best-fitting model, a single shift model is the next best-fitting (BF = 1.38). The most frequent model selected was the null model of homogeneous diversification (frequency = 0.85), this is due to the majority of BAMM runs not detecting any significant rate shifts (Additional file 8). Posterior distribution detected a model which had a diversification shift on an ancestral lineage (Additional file 8), though this model was substantially less frequent (frequency = 0.1).

### Body size evolution

The observed minimal morphological disparity through time is consistent with a non-adaptive diversification during the clades’ history. No significant disparity (95% CI) was detected in the clades’ history, although relatively high disparity is shown in the recent past (~ 1 Mya to present) indicating recent phenotypic divergence. The DTT plot (Fig. 2) showed consistency with the BM null model throughout most of the lineage’s history. Disparity of body size within clades is equal to or slightly higher than between clades (MDI = 0.113), but deviation from the null is not significant (*p* = 0.184). The positive MDI value is characteristic of species slightly overlapping in the body size morphospace. Reconstruction of species ancestral body size under BM on the phylogeny shows homogenous body size evolution with all species being morphologically similar (Fig. 2). Although a subclade (hereafter referred to as subclade A, see Fig. 2) with a common ancestor ~ 5 Mya had divergent body size evolution in the recent past. Other species that show extreme trait values diverged from a common ancestor in the distant past (*N. ocellatus* and *N. thierryi*).

**Fig. 3:**
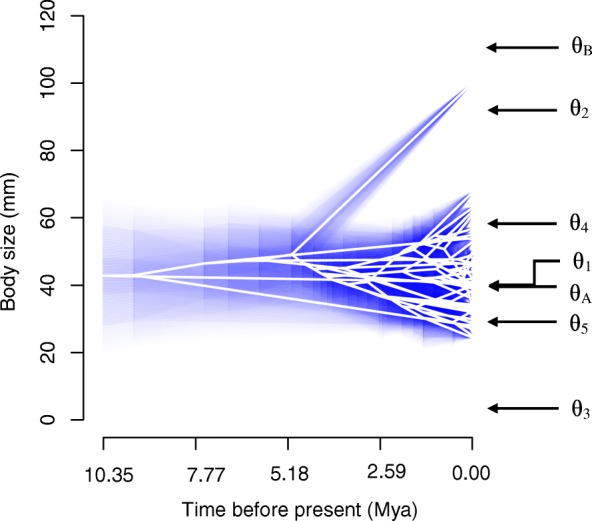
Phenogram of trait evolution of each lineage reconstructed under a Brownian motion model, with shading around lineages the 95% CI. Fitness optima (θ) under a multi-optima OU model shown on y-axis. θ_A_ and θ_B_ are optima from ℓ1ou, and θ_1_, θ_2_, θ_3_, θ_4_, and θ_5_ are optima from SURFACE

The OU model had the best-fit in continuous trait analysis, with a strong pull (α = 0.272). The Delta model of evolution comprehensively explains the pattern of the data, while BM showed marginal explanation, and EB was substantially incongruent (Table 2). The selection of OU over BM suggests there is a stabilizing selection pressure present around to one or more adaptive optima and falsifying a random walk mode of trait evolution. The greater likelihood of the Delta model of trait evolution over BM and EB – with a δ value more than 1 – suggests an adaptive proliferation late in the clades’ history. Stabilizing selection under an OU model would drive species to overlap in the morphospace around body size fitness optima. The two approaches to detect multiple OU optima identified different numbers of shifts. Both detected a shift on the terminal branch to *N. ocellatus*, with good bootstrap support from ℓ1ou (0.709). SURFACE detected this shift but also detected an additional four shifts on the adaptive landscape (Fig. 3). The two adaptive optima ℓ1ou detected were θ_A_ = 40.25 mm and θ_B_ = 115.79 mm, whereas SURFACE detected four adaptive optima within the body size range (θ_1_ = 41.26 mm, θ_2_ = 95.58 mm, θ_4_ = 59.74 mm, and θ_5_ = 30.57 mm) along with an optimum distant from all body sizes (θ_3_ = 4.76 mm). SURFACE found convergence between two species (*N. ocellatus* and *N. orthonotus*). Similar body size across many species is evidence of a stabilizing selection pressure, proposed in the OU model of trait evolution. Divergence was detected at three nodes of the phylogenetic tree all within subclade A using surface, while ℓ1ou did not detect any shifts in this subclade (Additional file 9).

**Table 2:** Continuous trait evolution analysis, using bias-corrected Akaike Information Criterion for model selection: using Brownian motion, Ornstein-Uhlenbeck, Early-Burst, and Delta models

Model	Model Parameters	σ^2^	InL	AICc	ΔAICc
Brownian Motion		0.027	−4.53	13.32	3.16
Ornstein-Uhlenbeck	α = 0.271885	0.049	−1.81	10.16	0
Early-Burst	α = −0.000001	0.027	−4.53	15.60	5.44
Delta	δ = 2.999999	0.012	−2.17	10.88	0.72

After ensuring MCMC convergence (likelihood ESS = 1801.00, number of shifts ESS = 1475.34), BAMM analysis of trait evolution found that the null model of no rate shifts of body size evolution was the best-fitting, as all other models had BF < 3 (Additional file 10). Although the posterior distribution found several models which include a single rate shift, the most congruent is the null model with trait evolution not undergoing significant shifts. The analysis detected four shifts all occurring in subclade A (Additional file 10).

### Trait-dependent diversification

Trait evolution analysis showed that OU is the best-fit model for *Nothobranchius* body size with a relatively strong pull (Table 2), thus the OU null model for *ES-sim* is the most appropriate to test. The OU null model showed diversification is not dependent on body size when testing for a monotonic relationship (*P* = 0.963), with minimum correlation between speciation rate and trait (Spearman’s rho = − 0.014; Additional file 11). Under a Brownian null model, diversification also showed insignificant trait-dependent diversification showing an equal relationship (Spearman’s rho = − 0.014, *P* = 0.957). QuaSSE analysis distinguished the modal model of diversification under stochastic trait evolution to best support the phylogenetic diversification with regards to body size (Table 3). Identifying body size to significantly affect the diversification of a clade (*p*≪0.05), inferring that species with intermediate body size have the highest diversification rate. Even though all other models display significance they show poorer support relative to the modal model (ΔAIC> 0). The models of trait-dependent diversification under the directional function deteriorated the goodness-of-fit of all models, indicating stochastic trait evolution.

**Table 3:** Quantitative State Speciation and Extinction (QuaSSE) analysis for body size dependency on diversification. Linear, Sigmoidal and Modal models were used

Model	InL	X^2^	P	AIC	ΔAIC
Linear	−131.16	5.2715	0.0216777	270.32	8.30
Sigmoidal	− 128.12	11.3579	0.0099401	268.24	6.22
Modal	−125.01	17.5754	0.0005381	262.02	0.00
Linear (φ)	−130.40	6.7934	0.0334840	270.80	8.78
Sigmoidal (φ)	−127.91	11.7817	0.0190503	269.81	7.79
Modal (φ)	− 124.91	17.7756	0.0013651	263.82	1.80

The models were run without and then with a directional function (indicated here by phi, *φ)*. *P* value to test significant difference to a model of constant speciation and extinction. Delta AIC (ΔAIC) calculated by comparing model to the best-fit, lowest AIC, model

### Biogeography of diversification

Our analyses comparing diversification patterns between the hotspot in lowland Tanzania and the surrounding areas (Fig. 1) that host low species diversities revealed the null, geography-independent model fitted the data best (Table 4). The single hidden state character-independent model (CID GeoSSE) was the best-fit, while the GeoSSE and CID GeoHiSSE models had the next best fit (ΔAICc> 9). These models can be interpreted to highlight the importance of non-biogeographic characters in diversification rate heterogeneity, and thus particular biogeographic regions are not driving diversification. Parameter estimates attained from model-averaging using AICw found that widespread species residing both in and out of the hotspot have the highest diversification rate (0.675 Sp/My) compared to species in the hotspot (0.265 Sp/My) and outside the hotspot (0.410 Sp/My).

**Table 4:** Geographic and hidden state speciation-extinction (GeoHiSSE) analysis for biogeographic region effect on diversification

Model	Hidden classes	InL	AICc	ΔAICc	AICw
GeoSSE	1	−119.246	255.225	9.952	0.004
GeoHiSSE	2	−114.635	273.815	28.542	1.45 × 10^−3^
CID GeoSSE	3	−112.836	245.273	0	0.962
CID GeoHiSSE	5	−112.202	257.070	11.797	0.033

Models run were: GeoSSE model dependent on geographic area; GeoHiSSE model dependent on geographic area and one hidden state; CID GeoSSE model independent of geography with hidden states null model for GeoSSE; CID GeoHiSSE model independent of geography with hidden states null model for GeoHiSSE

## Discussion

Our study provides supportive evidence for the hypothesis that the radiation of *Nothobranchius* killifish, a clade of exceptionally short-lived vertebrates, has proliferated into over 70 species with minimal niche diversification among them, thus relying on the availability of ‘spatial opportunity’ for species accumulations over time. Interestingly, diversification rates are lower in the one area in which multiple *Nothobranchius* species coexist (i.e., a species-richness hotspots), suggesting a potential slowdown of the speed of speciation events as species accumulate in the same geographic area. We argue that this link reinforces the view that diversification mediated by availability of ‘spatial opportunity’ is likely to be the dominant engine for *Nothobranchius* proliferation, relative to diversification mediated by niche divergence (i.e., adaptive radiation). Lineage diversification is coupled with body size evolution, which was also found to be homogeneous through time, with no rapid body size evolution at any point within the genus’ phylogenetic history. Body size disparity is minimal as all species except one are evolving under one adaptive regime.

### Tempo and mode of the Nothobranchius radiation

The niche-filling nature of adaptive radiations predicts a diversity-dependent signature of species accumulation in phylogenies. Whereas, diversification via non-adaptive radiation has more relaxed predictions about the diversification dynamics, which can range from early to late bursts of species accumulation. As we suggest above, as long as ‘spatial opportunity’ is available, species will encounter the conditions to radiate allopatrically [15, 18]. For example, the tempo of two non-adaptive radiations – *Plethodon* salamanders [8, 25] and *Rattus* rats [26] – have shown an early burst of species accumulation with a diversity-dependent decline, while another non-adaptive clade – *Phymaturus* lizards [18] – showed a late burst. Our study reveals a constant, diversity-independent rate of diversification over time without evidence for a slow-down. In addition, when species nested in the phylogeny access abundant spatial opportunity they may diversify faster than the sister lineages. We find no evidence of diversification rate heterogeneity, which again supports gradual access to suitable niche space for diversification in allopatry. However, the power to detect rate heterogeneity in a phylogeny of this size is low [84], especially when only few species may be diversifying under a different macroevolutionary regime [85, 86]. The savannah pools replicate the insular biogeographic conditions of archipelago systems and thus, impose dispersal limitations, and so diversification is due to extrinsic factors that promote allopatry (i.e. vicariance or flooding events, [37]). The widespread geographic range of *Nothobranchius* species indicates that there are environments with viable niches across East Africa. If dispersal was autonomous, as in terrestrial non-adaptive radiations [8, 25, 26], an early burst may take place as spatial opportunity could be accessed early in the clades’ history with a diversity-dependent decline. We argue that non-adaptive radiations cannot be determined based on the tempo of diversification alone; instead, criteria should include niche conservatism and phenotypic stasis.

*Nothobranchius* species have fundamentally diversified in allopatry, a characteristic of non-adaptive radiations, as confinement to micro-lacustrine environments is unconducive to sympatric speciation [15, 37, 38, 87, 88]. Stochastic environmental events that produce allopatric populations will likely have promoted diversification. East African major climatic fluctuations have taken place during *Nothobranchius’* history (~ 10 Mya to present) [89]. The establishment of Africa’s savannah in the late-Miocene (8–9 Mya) would have produced an abundance of spatial opportunity for the killifish, which develop via embryonic diapause [90–92] and can cope with seasonal droughts. Environmental fluctuations from 5 Mya to 1 Mya would have produced vicariance and may explain the presence of several sister species arising around this time [93]. These environmental drivers of diversification remain reasonable mechanisms for alternative dating of *Nothobranchius* diversification, starting either approximately ~ 14 Mya [94] or, as suggested by Furness et al. [95] even at around 50 Mya. Once allopatry is established speciation without ecological divergence potentially occurs via intense genetic drift in found populations without gene flow, driving reproductive isolation [38]. Alternatively, *Nothobranchius* exhibit a diverse array of karyotypes, therefore chromosomal speciation may cause speciation and may prevent hybridization upon secondary contact [96]; this process is detected in another non-adaptive radiation [26].

### Phenotypic evolution

Phenotypic evolution is the result of the selection pressures from biotic and abiotic interactions. In view of that fact, the stasis or disparity of a species’ body size will show how it is interacting with ecological space (niche) [44]. Body size disparity was consistent with variance expected under BM evolution for all of the genus’ history. Phylogenetic niche conservatism across the geographic range resulted in an OU process, with low disparity a result of evolution towards two certain adaptive optima, while additional optima may exist on the adaptive landscape. The close proximity of three peaks from SURFACE may be variance around a single peak, around which 47 species located, or the result of different diet at each peak, as size is a factor in diet composition [97–99]. Indeed, a common coexistence of three species often matches the three peaks in body size [45]. We acknowledge, however, that the reliability of the OU model from extant taxa data is questionable as over complex models are selected with high type I error in small trees [76, 100]; furthermore ancestral reconstruction under BM when trait is evolving under an OU model can produce misleading disparity [101]. A single species (*N. ocellatus*) was found to have largely divergent body size, evolving into predatory species that preys on smaller *Nothobranchius*. This evolutionary transition has not produced further divergence and *N. ocellatus* possesses relative wide geographic distribution. Interestingly, in another clade of annual killifish from southern Neotropics, *Austrolebias*, the same evolutionary transition has led to a group of five predatory species. Whether they all are the result of a single evolutionary transition with consequent diversification into allopatric species [102] or evolved repeatedly [103, 104], is unresolved. Apart from the rare evolution of gigantism, there is relatively low morphological and ecological disparity in killifish; with similar niche space occupied by African and South American genera [42, 105]. Thus, it can be predicted that killifish’s dependency on a specific microhabitat, due to their life history adaptation of annualism, has constrained their ability to exploit ecologically different niche space.

Interestingly, the spatial overlap among the species in the hotspot that our study has identified is hypothesised to have promoted divergence in body size via ecological character displacement to reduce competition for niche space in the spatially restrictive pools, a process that has been identified in *Nothobranchius* species under certain conditions [98]. Seasonal pools in costal Tanzania, in particular, support coexistence of species that are spatially separated between very shallow marginal habitats and deeper water, while their feeding morphology also suggests some level of trophic differentiation [42]. Most small-bodied species from coastal Tanzania were not included in the *Nothobranchius* phylogeny [37], hindering insights into the evolutionary transitions to their functional niche. Frequent flooding in coastal regions of Tanzania would prove a viable mode of dispersal from which sympatry is established from secondary contact [106]. Therefore, once secondary contact is established, divergent natural selection could drive body size diversification. As divergent evolution of body size is only strongly supported in one species and not repeatedly found in allopatric species, it is unlikely that the divergent morphology of sympatric species occurs prior to secondary contact.

### The phenotypic and biogeographic nature of diversification

The hypothesis that variation in body size influences variation in diversification rates is rejected by our analyses, although results provided contradictory outcomes. Speciation was found to not show a linear or sigmoidal relationship with body size with *ES-sim,* while QuaSSE analysis found both models as well as a modal model to have a significant relationship with diversification. We accept the results of *ES-sim*, due to its lower type I error [70], and as body size is predicted to be unimportant when diversification is dependent on abiotic factors (i.e. flooding). GeoHiSSE parameter estimates showed that diversification rate within the hotspot is lower than in the surrounding regions, finding diversification has not the caused the high density of species [107]. The higher species richness is potentially linked to the frequent flooding which increases dispersal into the hotspot but negates divergence through gene flow when populations are reconnected compared to inland Africa where flooding, and connection between suitable habitats is less frequent [32]. The East African rift system uplift caused increased rainfall in inland Tanzania, with coastal regions near Dar es Salaam frequented by flooding [106, 108]. Hidden states allow for adequate testing of the GeoSSE model, however, although diversification rate heterogeneity is found to be minimal the inability to model continuous characters with hidden states leaves the QuaSSE model vulnerable to a high type I error rate, as well as the limited power of SSE models on small phylogenies [109–112].

## Conclusion

Our study investigates the macroevolution of a non-adaptive radiation model system – the African *Nothobranchius* killifish. The genus has diversified at a constant rate through time due to gradual exploitation of spatial (rather than ecological) opportunity. The evolution of embryo diapause in this continental genus, driven by natural selection arising from the desiccation of their habitats every year, has facilitated their diversification across eastern Africa. However, we suggest that this life history trait has constrained *Nothobranchius* and other killifish adaptively, most likely due to the substrate dependency of alluvial vertisol soils. Therefore, variance in phenotypic evolution has been relatively constrained as ancestral niche space is conserved from diversification via spatial opportunity, and thus phenotypic disparity is minimal. The exception of a hotspot of species richness which displays higher body size diversity indicates that under certain conditions *Nothobranchius* may be able to diverge phenotypically. Collectively, our study contributes to the accumulating evidence about a process of evolution that results in the (sometimes) prolific diversification of lineages in the absence of niche-filling, and thus, in the absence of adaptive niche diversification.

## Additional files

Additional file 1: List of references from which the body size data is taken. (DOCX 15 kb)

Additional file 2: Body size data of 48 *Nothobranchius* species used in this study. (PDF 35 kb)

Additional file 3: List of references from which locality data is taken. (PDF 4 kb)

Additional file 4: Geographic locality data for 49 *Nothobranchius* species corresponding to the species in the phylogeny. (DOCX 18 kb)

Additional file 5: Nothobranchius phylogeny (*n* = 49) with colours representing geographic region; blue: endemic to hotspot, red: widespread, green: non-endemic to hotspot. (DOCX 16 kb)

Additional file 6: (A) The distribution of logarithms of likelihood ratio of constant rate Birth-Death (crBD) and (B) diversity-dependent diversification linear speciation and extinction (DDL + E) from parametric bootstrapping. Both models were run with 22 missing species and a significance value (α) set to 0.05. The blue arrow shows the logarithm of likelihood ratio for the significance value (2.99). The black arrow shows the logarithm of likelihood ratio for the data (0.12). (DOCX 31 kb)

Additional file 7: (A) The distribution of logarithms of likelihood ratio of constant rate Birth-Death (crBD) and (B) diversity-dependent diversification linear speciation and extinction (DDL + E) from parametric bootstrapping. Both models were run with 50 missing species and a significance value (α) set to 0.05. The blue arrows is the logarithm ratio for the significance value (3.07). The black arrow shows the logarithm of likelihood ratio for the data (0.10). (PDF 27 kb)

Additional file 8: Phylogenetic tree with speciation rate shown by colour gradient. Black dot depict significant shifts in diversification rate. Frequency (f) of the model selected show above respective model. (PDF 181 kb)

Additional file 9: Left, ℓ1ou selective regimes, red line indicates divergence to a different selective regime, with asterisk and strength of shift on the node. Right, SURFACE selective regimes plotted regime shift are numbered on nodes, regimes are differentiate by colour, grey is divergence and red is convergence, (1) is the ancestral regime and thus is not shown on the tree. (PDF 182 kb)

Additional file 10: Phylogenetic tree with rate of phenotypic evolution shown by colour gradient. Black dots show significant shifts in trait evolution and frequency (f) of model selected show above respective model. (PDF 22 kb)

Additional file 11: Scatter plot of the logarithm of the speciation rate against the body size of *Nothobranchius* species. Speciation rate is measured using the ES-method. (PDF 36 kb)

## Abbreviations

AIC: Akaike Information Criterion
AICc: Bias-corrected AIC
BAMM: Bayesian Analysis of Macroevolutionary Mixtures
BF: Bayes factors
BM: Brownian motion
crBD: constant rate birth-death
DDE + E: diversity-dependent exponential
DDL + E: diversity-dependent linear
DTT: disparity through time
EB: early-burst
ESS: effective sample size
ES-sim: equal-splits with simulated null model
GeoSSE: Geographic State Speciation-Extinction
LTT: lineage through-time
MCCR: Monte Carlo Constant Rate
MCMC: Markov Chain Monte Carlo
MDI: morphological disparity index
Mya: million years ago
OU: Ornstein-Uhlenbeck
QuaSSE: Quantitative State Speciation Speciation and Extinction
SL_max_: maximum standard length
Sp/My: Species per million years
