# Supplementary material for: Live fast, diversify non-adaptively: evolutionary diversification of exceptionally short-lived annual killifishes
Source: BMC Evol Biol. 2019 Jan 9;19:10. doi: 10.1186/s12862-019-1344-0 (PMC6327596; doi:10.1186/s12862-019-1344-0)
Supplement: Supplementary file 1 — List of references from which the body size data is taken. (DOCX 15 kb) [file 12862_2019_1344_MOESM1_ESM.docx]

**Additional file 1**

**List of references from which the body size data is taken**

Ng'oma, E., S. Valdesalici, K. Reichwald and A. Cellerino, 2013. Genetic and morphological studies of Nothobranchius (Cyprinodontiformes) from Malawi with description of Nothobranchius wattersi sp. nov. J. Fish Biol. 82:165-188.

Reichard, M., 2010. Nothobranchius kadleci (Cyprinodontiformes: Nothobranchiidae), a new species of annual killifish from central Mozambique. Zootaxa 2332:49-60.

Seegers L. Killifishes of the world. Old world killis II. Aqualog. Verlag A.C.S. GmbH

Valdesalici S. 2014. *Nothobranchius bellemansi* and *Nothobranchius occultus* (Cyprinodontiformes: Nothobranchiidae) two new annual killifish from Sudan. Killi-Data Series 2014: 4-19.

Valdesalici, S. and G. Amato, 2011. Nothobranchius oestergaardi (Cyprinodontiformes: Nothobranchiidae), a new annual killifish from Mweru Wantipa Lake drainage basin, northern Zambia. Aqua, Int. J. Ichthyol. 17(2):111-119.

Valdesalici, S. and K. Kardashev, 2011. Nothobranchius seegersi (Cyprinodontiformes: Nothobranchiidae), a new annual killifish from the Malagarasi River drainage, Tanzania. Bonn Zool. Bull. 60(1):89-93.

Valdesalici, S. and R.H. Wildekamp, 2004. A new species of the genus Nothobranchius Peters, 1868 from the Lufwa River basin, Katanga Province, Democratic Republic of Congo (Pisces, Cyprinodontiformes, Aplocheilidae). Ann. Mus. Civ. Stor. Nat. "G. Doria" 96:241-251.

Valdesalici, S., 2007. A new species of the genus Nothobranchius (Cyprinodontiformes: Nothobranchiidae) from the coastal area of northeastern Mozambique. Zootaxa 1587:61-68.

Valdesalici, S., 2010. Nothobranchius boklundi (Cyprinodontiformes: Nothobranchiidae): a new annual killifish with two male colour morphs from the Luangwa River basin, Zambia. Aqua, Int. J. Ichthyol. 16(2):51-60.

Valdesalici, S., M. Bellemans, K. Kardashev and A. Golubtsov, 2009. Nothobranchius nubaensis (Cyprinodontiformes: Nothobranchiidae) a new annual killifish from Sudan and Ethiopia. Aqua, Int. J. Ichthyol. 15(3): 143-152.

Valdesalici, S., R. Bills, A. Dorn, K. Reichwald and A. Cellerino, 2012. Nothobranchius niassa (Cyprinodontiformes: Nothobranchiidae), a new species of annual killifish from northern Mozambique. Ichthyol. Explor. Freshwat. 23(1):19-28.

Watters BR, Wildekamp RH, Shidlovskiy KM. 2014. Description and biogeography of *Nothobranchius capriviensis*, a new species of annual killifish from the Zambezi Region of Namibia (Cyprinodontiformes: Nothobranchiidae). JAKA 47(4–6): 97–133.

Watters, B.W., B.J. Cooper and R.H. Wildekamp, 2008. Description of Nothobranchius cardinalis spec. nov. (Cyprinodontiformes: Aplocheilidae), an annual fish from the Mbwemkuru River basin, Tanzania. J. Am. Killifsh Ass. 40(5&6):129-145.

Wildekamp RH. A world of killies: atlas of the oviparous cyprindontiform fishes of the world. 4th Edition. Elyria; American Killifish Association; 2004.

Wildekamp, R.H., K.M. Shidlovskiy and B.R. Watters, 2009. Systematics of Nothobranchius melanospilus species group (Cyprinodontiforme: Nothobranchidae) with description of two new species from Tanzania and Mozambique. Ichthyol. Explor. Freshwat. 20(3):237-254.
