# Supplementary figures and images for: Live fast, diversify non-adaptively: evolutionary diversification of exceptionally short-lived annual killifishes
Source: BMC Evol Biol. 2019 Jan 9;19:10. doi: 10.1186/s12862-019-1344-0 (PMC6327596; doi:10.1186/s12862-019-1344-0)

$f = 0.67$

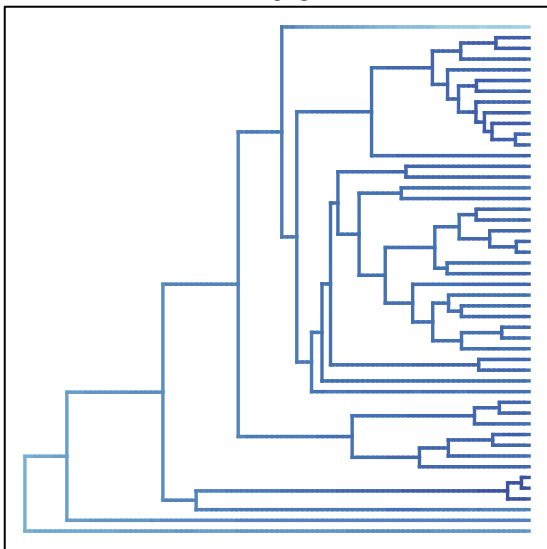

$f = 0.11$

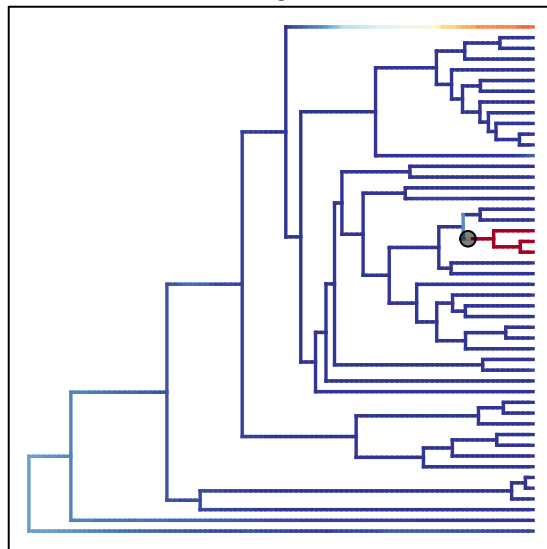

$f = 0.089$

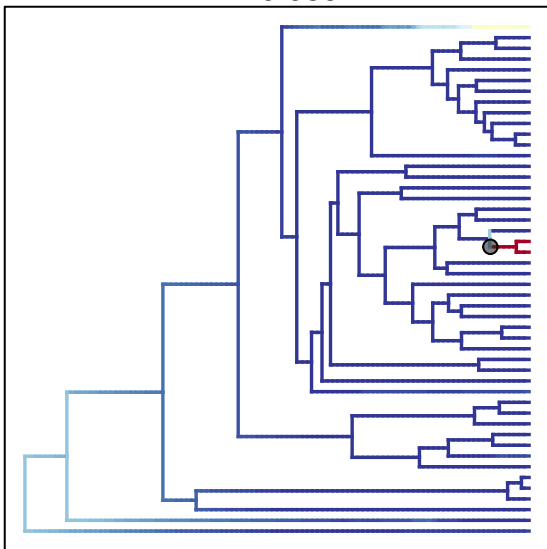

$f = 0.073$

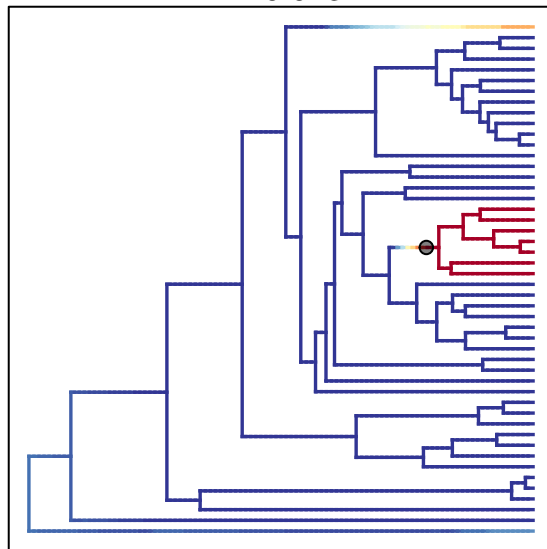

Supplement: Supplementary file 2 — Body size data of 48 Nothobranchius species used in this study. (PDF 35 kb) [file 12862_2019_1344_MOESM2_ESM.pdf]

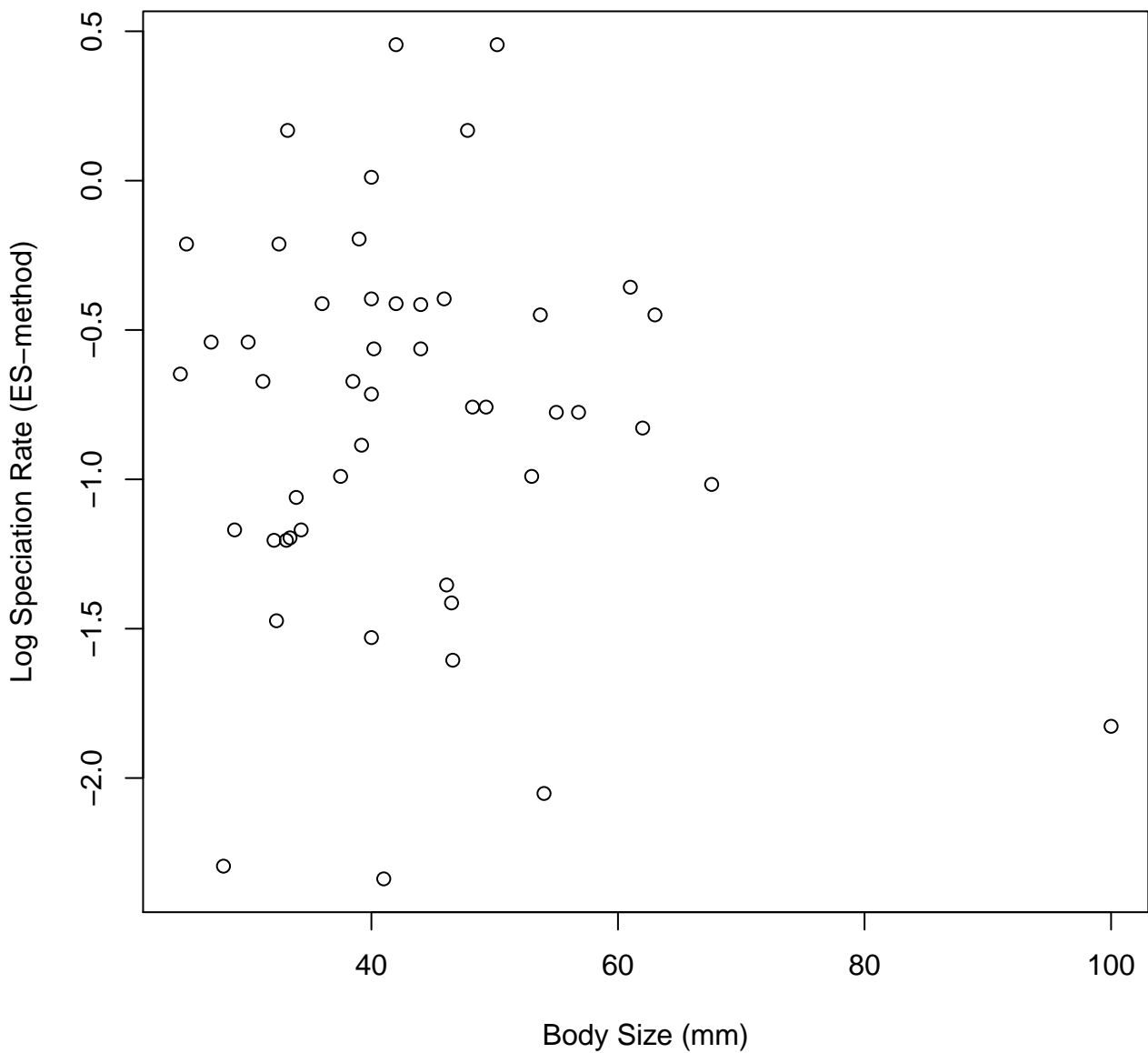

Supplement: Supplementary file 3 — List of references from which locality data is taken. (PDF 4 kb) [file 12862_2019_1344_MOESM3_ESM.pdf]

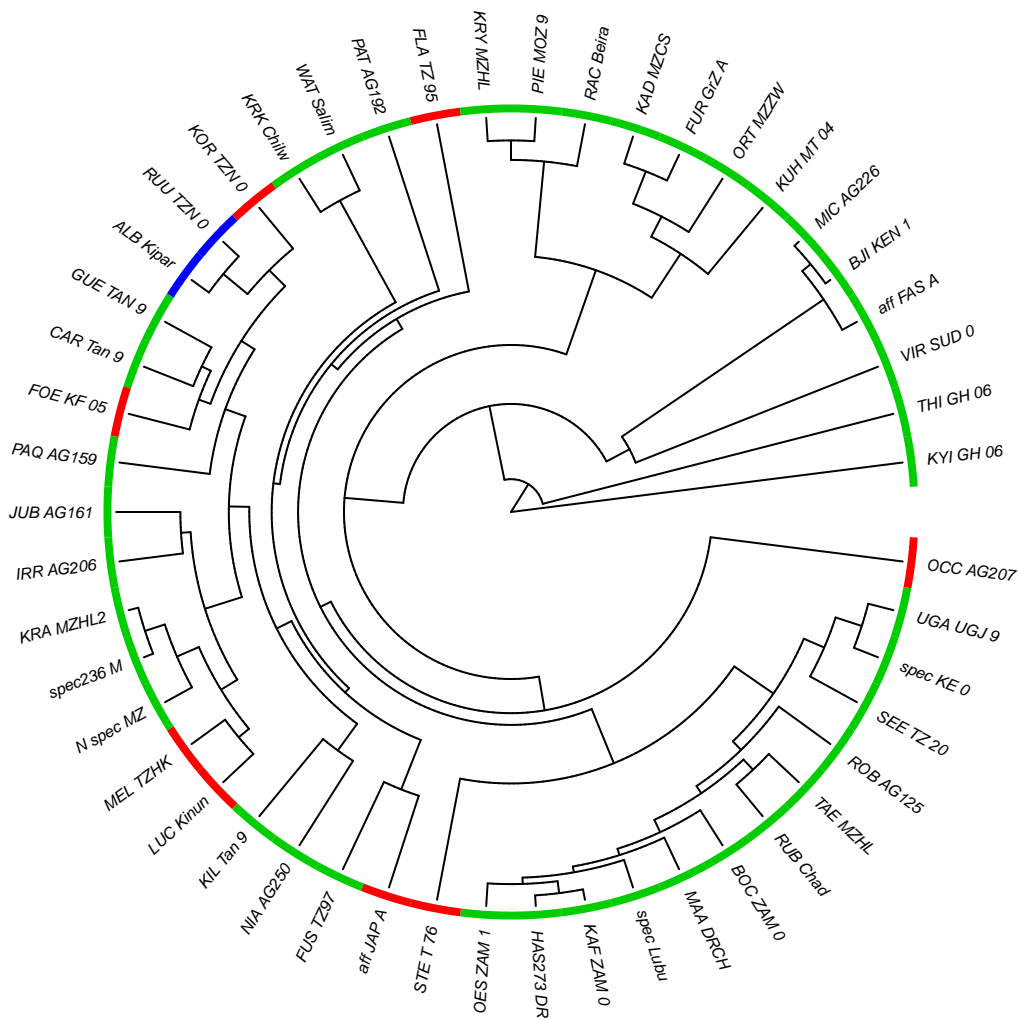

Supplement: Supplementary file 7 — (A) The distribution of logarithms of likelihood ratio of constant rate Birth-Death (crBD) and (B) diversity-dependent diversification linear speciation and extinction (DDL + E) from parametric bootstrapping. Both models were run with 50 missing species and a significance value (α) set to 0.05. The blue arrows is the logarithm ratio for the significance value (3.07). The black arrow shows the logarithm of likelihood ratio for the data (0.10). (PDF 27 kb) [file 12862_2019_1344_MOESM7_ESM.pdf]

(A)

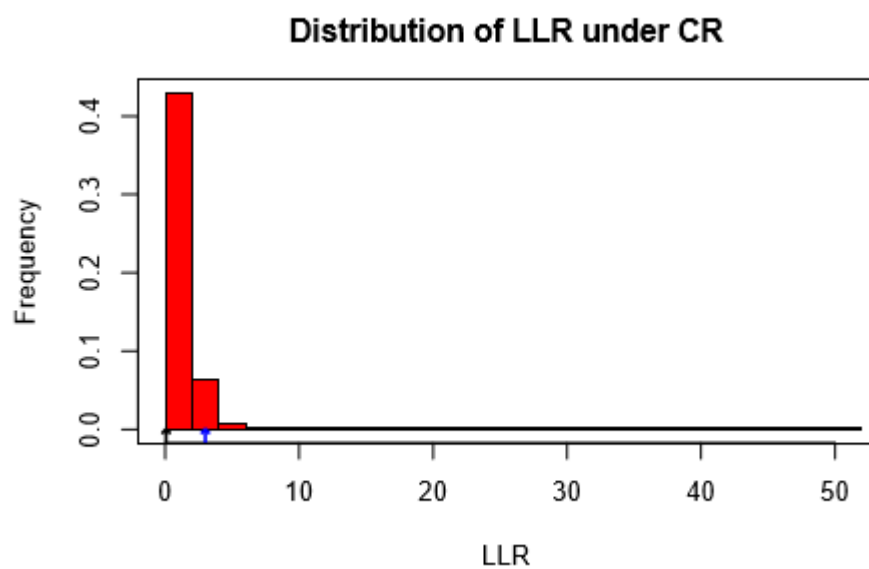

(B)

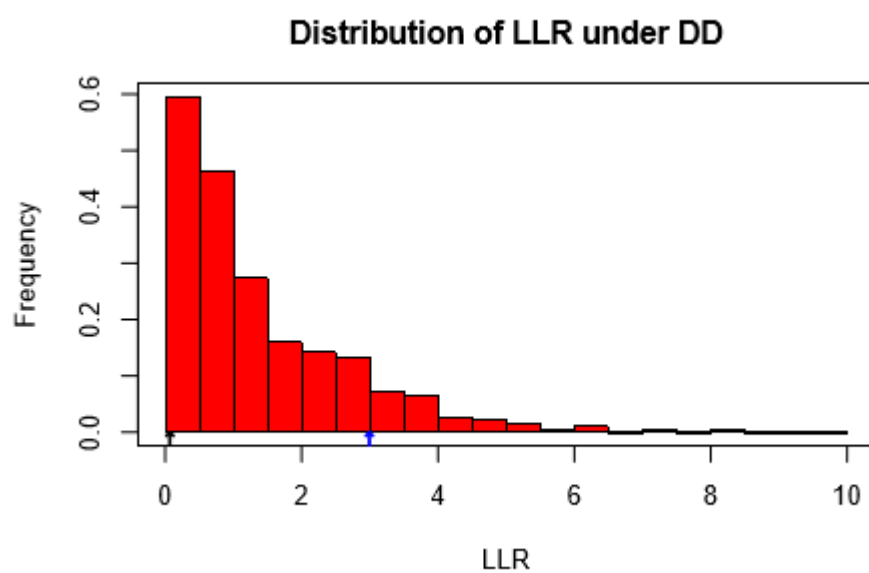

Supplement: Supplementary file 8 — Phylogenetic tree with speciation rate shown by colour gradient. Black dot depict significant shifts in diversification rate. Frequency (f) of the model selected show above respective model. (PDF 181 kb) [file 12862_2019_1344_MOESM8_ESM.pdf]

(A)

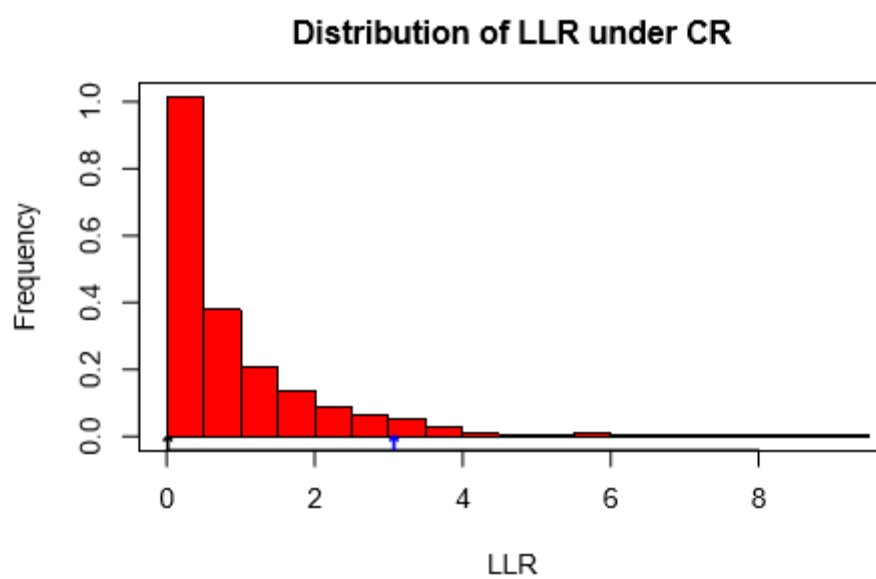

(B)

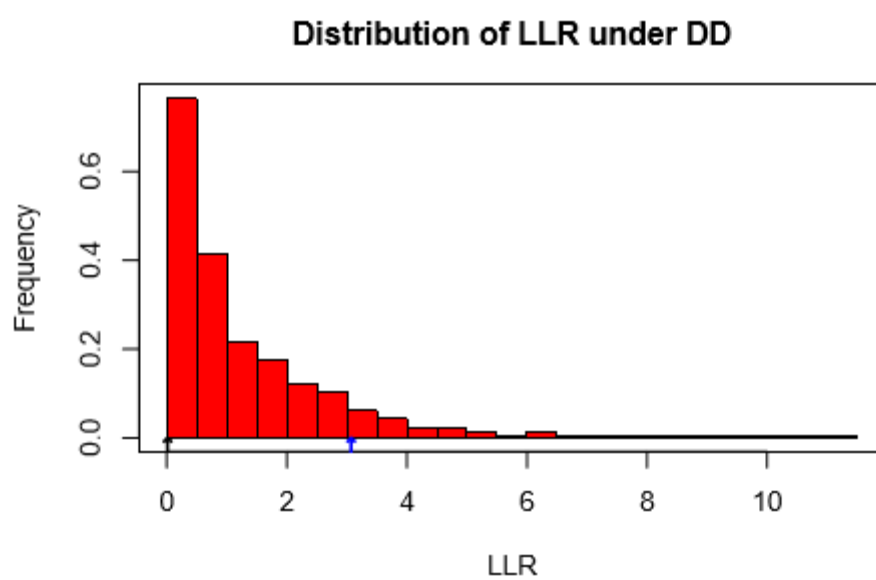

Supplement: Supplementary file 9 — Left, ℓ1ou selective regimes, red line indicates divergence to a different selective regime, with asterisk and strength of shift on the node. Right, SURFACE selective regimes plotted regime shift are numbered on nodes, regimes are differentiate by colour, grey is divergence and red is convergence, (1) is the ancestral regime and thus is not shown on the tree. (PDF 182 kb) [file 12862_2019_1344_MOESM9_ESM.pdf]

$f = 0.85$

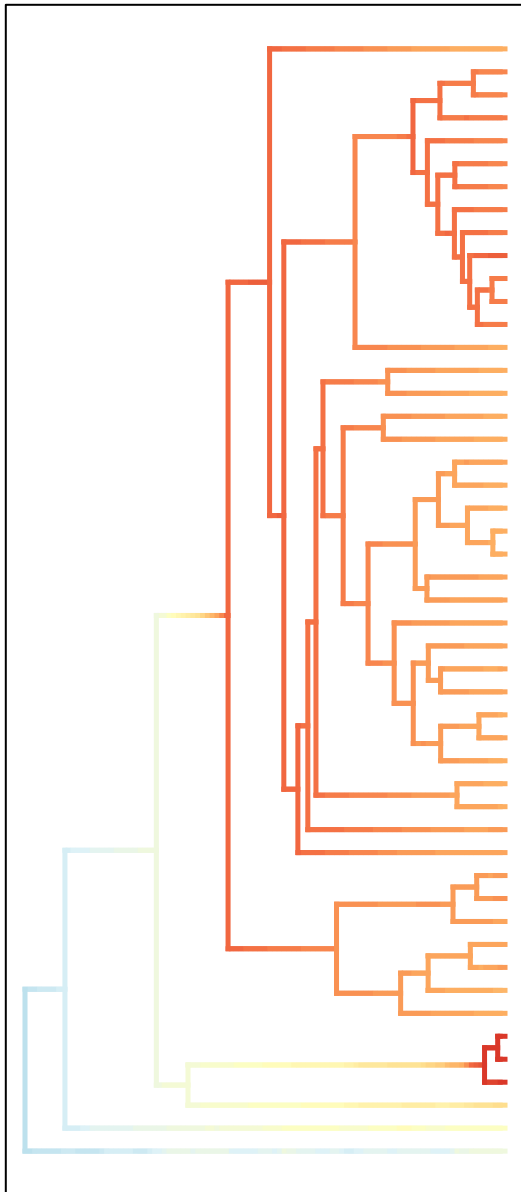

$f = 0.1$

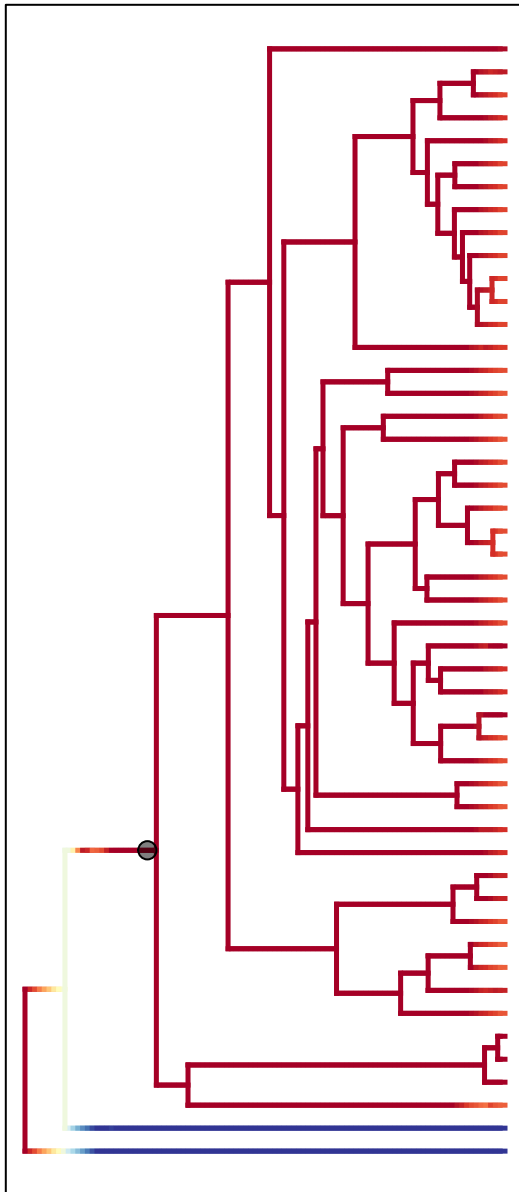

Supplement: Supplementary file 10 — Phylogenetic tree with rate of phenotypic evolution shown by colour gradient. Black dots show significant shifts in trait evolution and frequency (f) of model selected show above respective model. (PDF 22 kb) [file 12862_2019_1344_MOESM10_ESM.pdf]

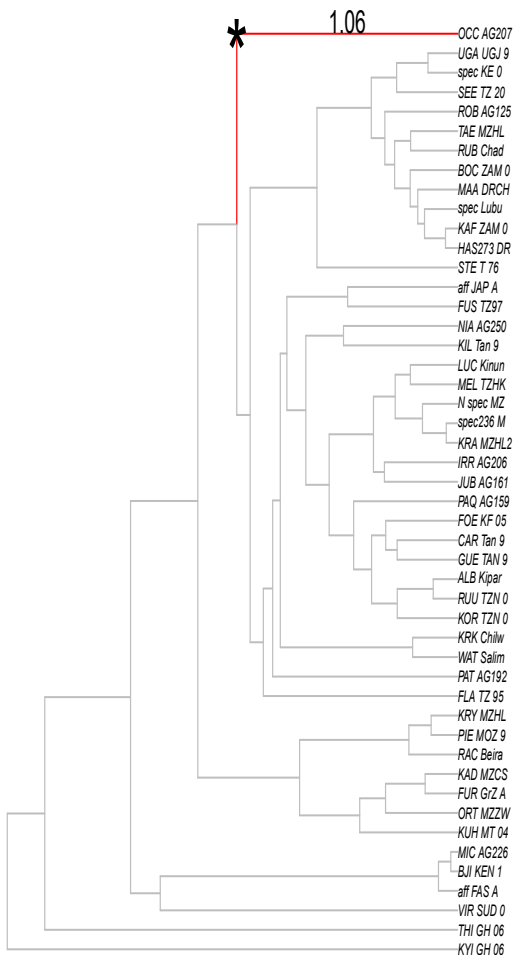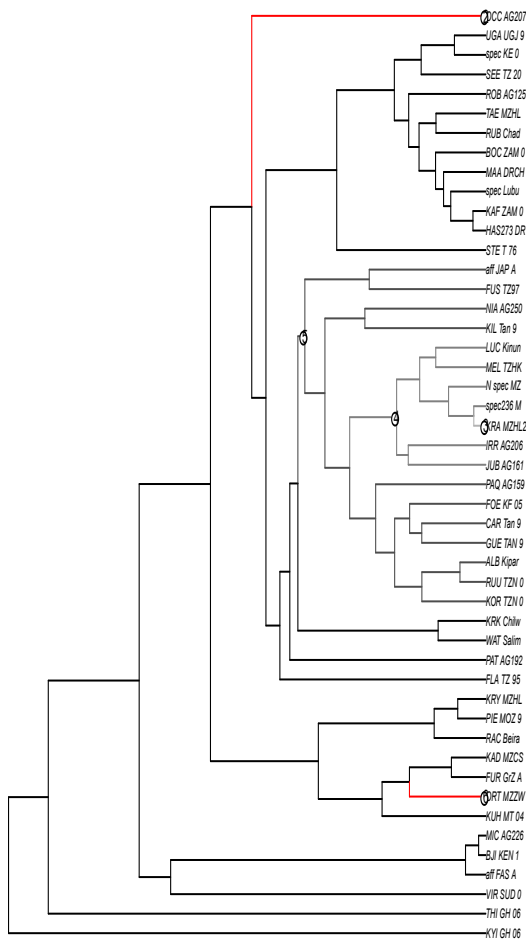

Supplement: Supplementary file 11 — Scatter plot of the logarithm of the speciation rate against the body size of Nothobranchius species. Speciation rate is measured using the ES-method. (PDF 36 kb) [file 12862_2019_1344_MOESM11_ESM.pdf]
