# Supplementary material for: Live fast, diversify non-adaptively: evolutionary diversification of exceptionally short-lived annual killifishes
Source: BMC Evol Biol. 2019 Jan 9;19:10. doi: 10.1186/s12862-019-1344-0 (PMC6327596; doi:10.1186/s12862-019-1344-0)
Supplement: Supplementary file 4 — Geographic locality data for 49 Nothobranchius species corresponding to the species in the phylogeny. (DOCX 18 kb) [file 12862_2019_1344_MOESM4_ESM.docx]

| Phylogeny name | Body size (mm) | In(body size) |
| --- | --- | --- |
| ALB_Kipar | 32.5 | 3.481240089 |
| BJI_KEN_1 | 48.2 | 3.875359021 |
| BOC_ZAM_0 | 44 | 3.784189634 |
| CAR_Tan_9 | 27 | 3.295836866 |
| aff_FAS_A | 46.5 | 3.839452313 |
| FLA_TZ_95 | 46.6 | 3.841600541 |
| FOE_KF_05 | 40 | 3.688879454 |
| FUR_GrZ_A | 44 | 3.784189634 |
| FUS_TZ97_ | 33.1 | 3.499533282 |
| GUE_TAN_9 | 30.0 | 3.401197382 |
| HAS273_DR | 42.0 | 3.737669618 |
| IRR_AG206 | 56.8 | 4.039536326 |
| aff_JAP_A | 32.1 | 3.46885603 |
| JUB_AG161 | 55.0 | 4.007333185 |
| KAD_MZCS_ | 40.2 | 3.693866996 |
| KAF_ZAM_0 | 50.2 | 3.916015027 |
| KIL_Tan_9 | 34.3 | 3.535145354 |
| KRK_Chilw | 37.5 | 3.624340933 |
| KOR_TZN_0 | 24.5 | 3.198673118 |
| KRA_MZHL2 | 33.2 | 3.502549876 |
| KRY_MZHL_ | 31.2 | 3.440418095 |
| KUH_MT_04 | 46.1 | 3.83081295 |
| LUC_Kinun | 63.0 | 4.143134726 |
| N_spec_MZ | 61.0 | 4.110873864 |
| MAA_DRCH_ | 39.0 | 3.663561646 |
| MEL_TZHK_ | 53.7 | 3.983413002 |
| MIC_AG226 | 49.3 | 3.897924081 |
| NIA_AG250 | 28.9 | 3.363841595 |
| OCC_AG207 | 100.0 | 4.605170186 |
| ORT_MZZW_ | 67.6 | 4.213607983 |
| PAQ_AG159 | 33.9 | 3.523415014 |
| PAT_AG192 | 40.0 | 3.688879454 |
| PIE_MOZ_9 | 38.5 | 3.650658241 |
| RAC_Beira | 33.4 | 3.5085559 |
| ROB_AG125 | 39.2 | 3.668676747 |
| RUB_Chad_ | 36.0 | 3.583518938 |
| RUU_TZN_0 | 25.0 | 3.218875825 |
| SEE_TZ_20 | 62.0 | 4.127134385 |
| STE_T_76_ | 32.3 | 3.47506723 |
| TAE_MZHL_ | 42.0 | 3.737669618 |
| THI_GH_06 | 28.0 | 3.33220451 |
| UGA_UGJ_9 | 45.9 | 3.826465117 |
| VIR_SUD_0 | 54.0 | 3.988984047 |
| WAT_Salim | 53.0 | 3.970291914 |
| KYI_GH_06 | 41.0 | 3.713572067 |
| spec_Lubu | 40.0 | 3.688879454 |
| spec236_M | 47.8 | 3.867025639 |
| spec_KE_0 | 40.0 | 3.688879454 |
