# Supplementary material for: Live fast, diversify non-adaptively: evolutionary diversification of exceptionally short-lived annual killifishes
Source: BMC Evol Biol. 2019 Jan 9;19:10. doi: 10.1186/s12862-019-1344-0 (PMC6327596; doi:10.1186/s12862-019-1344-0)
Supplement: Supplementary file 5 — Nothobranchius phylogeny (n = 49) with colours representing geographic region; blue: endemic to hotspot, red: widespread, green: non-endemic to hotspot. (DOCX 16 kb) [file 12862_2019_1344_MOESM5_ESM.docx]

**Additional file 3**

**Geographic locality data for 49 *Nothobranchius* species corresponding to the species in the phylogeny.**

Agnew M. 2011. Notes on *Nothobranchius*. JAKA 44(4): 124–127.

Nagy B, Kis AH. 2010. Variation in habitat characteristics and the occurrence of *Nothobranchius* species in seasonal biotopes of Tanzania. JAKA: 43(5): 130–144.

Nagy B, Shidlovskiy K. 2012. Phylogenetic relationships and biogeography of *Nothobranchius* species (Cyprinodontiformes: Nothobranchiidae) of coastal Kenya. JAKA 45(2): 34–51.

Nagy B. 2009. Distribution of *Nothobranchius* species in the coastal part of Kenya. JAKA 42(5): 194–214.

Nagy B. 2010. Collecting *Nothobranchius* in „the pearl of Africa“. Nothobranchius archives 1(1): 14–30.

Nagy B. 2010. In search of *Nothobranchius bojiensis* in the wilderness of northeastern Kenya. *Nothobranchius* archives 1(4): 3–17.

Nagy B. 2011. *Nothobranchius* explorations in the Nuba Mountains. JAKA 44(2): 37–57.

Nagy B. 2013. Five Locations – Sometimes it is not easy to re-collect *Nothobranchius* from the same locality. JAKA 46(6): 162–169.

Nagy B. 2015. More on the search for *Nothobranchius brieni*. JAKA 48(1–2): 3–24.

Ng'oma, E., S. Valdesalici, K. Reichwald and A. Cellerino, 2013. Genetic and morphological studies of Nothobranchius (Cyprinodontiformes) from Malawi with description of Nothobranchius wattersi sp. nov. J. Fish Biol. 82:165-188.

Schmidt O. 2002. Searching for killifish in Zambia. JAKA 35(1): 3–17.

Shidlovskiy K. 2010. Collecting *Nothobranchius* in South-Western Tanzania. Nothobranchius archives 1(4): 18–27.

Valdesalici S. 2014. *Nothobranchius bellemansi* and *Nothobranchius occultus* (Cyprinodontiformes: Nothobranchiidae) two new annual killifish from Sudan. Killi-Data Series 2014: 4-19.

Valdesalici S. 2014. The genus *Pronothobranchius*. JAKA 47(2&3): 74–79.

Valdesalici, S. and G. Amato, 2011. Nothobranchius oestergaardi (Cyprinodontiformes: Nothobranchiidae), a new annual killifish from Mweru Wantipa Lake drainage basin, northern Zambia. Aqua, Int. J. Ichthyol. 17(2):111-119.

Valdesalici, S. and K. Kardashev, 2011. Nothobranchius seegersi (Cyprinodontiformes: Nothobranchiidae), a new annual killifish from the Malagarasi River drainage, Tanzania. Bonn Zool. Bull. 60(1):89-93.

Valdesalici, S. and R.H. Wildekamp, 2004. A new species of the genus Nothobranchius Peters, 1868 from the Lufwa River basin, Katanga Province, Democratic Republic of Congo (Pisces, Cyprinodontiformes, Aplocheilidae). Ann. Mus. Civ. Stor. Nat. "G. Doria" 96:241-251.

Valdesalici, S., 2007. A new species of the genus Nothobranchius (Cyprinodontiformes: Nothobranchiidae) from the coastal area of northeastern Mozambique. Zootaxa 1587:61-68.

Valdesalici, S., 2010. Nothobranchius boklundi (Cyprinodontiformes: Nothobranchiidae): a new annual killifish with two male colour morphs from the Luangwa River basin, Zambia. Aqua, Int. J. Ichthyol. 16(2):51-60.

Van der Zee J. 2011. An introduction to killifishes of the Congo basin. JAKA 44(5): 142–160.

Watters BR, Wildekamp RH, Cooper BJ. 2009. *Nothobranchius rachovii* Ahl, 1926 – a historical review. JAKA 42(2): 105–127.

Watters BR, Wildekamp RH, Shidlovskiy KM. 2014. Description and biogeography of *Nothobranchius capriviensis*, a new species of annual killifish from the Zambezi Region of Namibia (Cyprinodontiformes: Nothobranchiidae). JAKA 47(4–6): 97–133.

Watters BR. 2006. A study of *Nothobranchius* habitats in the great Limpopo transfrontier park. JAKA 39(1): 14–25.

Watters BR. 2009. The ecology and distribution of *Nothobranchius* fishes. JAKA 42(2): 37–76.

Watters, B.W., B.J. Cooper and R.H. Wildekamp, 2008. Description of Nothobranchius cardinalis spec. nov. (Cyprinodontiformes: Aplocheilidae), an annual fish from the Mbwemkuru River basin, Tanzania. J. Am. Killifsh Ass. 40(5&6):129-145.

Wildekamp R. 2010. Early history of the genus *Nothobranchius*. Nothobranchius archives 1(1): 3–13.

Wildekamp RH. A world of killies: atlas of the oviparous cyprindontiform fishes of the world. 4th Edition. Elyria; American Killifish Association; 2004.

Wildekamp, R.H., K.M. Shidlovskiy and B.R. Watters, 2009. Systematics of Nothobranchius melanospilus species group (Cyprinodontiforme: Nothobranchidae) with description of two new species from Tanzania and Mozambique. Ichthyol. Explor. Freshwat. 20(3):237-254.
