# Supplementary material for: Live fast, diversify non-adaptively: evolutionary diversification of exceptionally short-lived annual killifishes
Source: BMC Evol Biol. 2019 Jan 9;19:10. doi: 10.1186/s12862-019-1344-0 (PMC6327596; doi:10.1186/s12862-019-1344-0)
Supplement: Supplementary file 6 — (A) The distribution of logarithms of likelihood ratio of constant rate Birth-Death (crBD) and (B) diversity-dependent diversification linear speciation and extinction (DDL + E) from parametric bootstrapping. Both models were run with 22 missing species and a significance value (α) set to 0.05. The blue arrow shows the logarithm of likelihood ratio for the significance value (2.99). The black arrow shows the logarithm of likelihood ratio for the data (0.12). (DOCX 31 kb) [file 12862_2019_1344_MOESM6_ESM.docx]

| Species | Longitude (x) | Latitude (y) |
| --- | --- | --- |
| Nothobranchius albimarginatus | 38.94 | -7.136667 |
| Nothobranchius boijensis | 39.166667 | 1.5 |
| Nothobranchius boklundi | 31.766667 | -13.1 |
| Nothobranchius cardinalis | 38.2564 | -9.9017 |
| Nothobranchius cardinalis | 38.25 | -9.9 |
| Nothobranchius cardinalis | 39.49605 | -9.57445 |
| Nothobranchius fasciatus | 40 | -1.45 |
| Nothobranchius fasciatus | 41.866667 | -0.716667 |
| Nothobranchius fasciatus | 41.594167 | -0.569167 |
| Nothobranchius fasciatus | 41.3275 | 0.2775 |
| Nothobranchius fasciatus | 41.698889 | 0.413333 |
| Nothobranchius flammicomantis | 37.716667 | -7.4 |
| Nothobranchius flammicomantis | 38.067222 | -7.368611 |
| Nothobranchius flammicomantis | 38.777778 | -6.585833 |
| Nothobranchius foerschi | 39.032778 | -6.729444 |
| Nothobranchius foerschi | 38.816667 | -6.716667 |
| Nothobranchius foerschi | 38.813056 | -6.544722 |
| Nothobranchius foerschi | 38.858889 | -6.4225 |
| Nothobranchius furzeri | 32.782222 | -24.411 |
| Nothobranchius furzeri | 32.975 | -24.35 |
| Nothobranchius furzeri | 32.976667 | -24.35 |
| Nothobranchius furzeri | 32.891667 | -24.3125 |
| Nothobranchius furzeri | 32.6125 | -24.3 |
| Nothobranchius furzeri | 33.176667 | -24.229167 |
| Nothobranchius furzeri | 32.837778 | -24.219444 |
| Nothobranchius furzeri | 32.829167 | -24.208333 |
| Nothobranchius furzeri | 33.325833 | -24.126667 |
| Nothobranchius furzeri | 32.716667 | -24.05 |
| Nothobranchius furzeri | 32.708333 | -24.041667 |
| Nothobranchius furzeri | 32.694444 | -23.960833 |
| Nothobranchius furzeri | 32.57195 | -23.4989 |
| Nothobranchius furzeri | 32.5671 | -23.474067 |
| Nothobranchius furzeri | 33.033267 | -22.319367 |
| Nothobranchius furzeri | 31.733333 | -21.822222 |
| Nothobranchius furzeri | 31.5975 | -21.726389 |
| Nothobranchius furzeri | 31.75 | -21.666667 |
| Nothobranchius fuscotaeniatus | 38.654444 | -8.020833 |
| Nothobranchius fuscotaeniatus | 39 | -3.216667 |
| Nothobranchius guentheri | 39.343889 | -6.130833 |
| Nothobranchius guentheri | 39.333333 | -6.033333 |
| Nothobranchius guentheri | 39.329444 | -6.011167 |
| Nothobranchius guentheri | 39.272778 | -5.918889 |
| Nothobranchius hassoni | 26.9655 | -10.415556 |
| Nothobranchius hassoni | 26.95 | -10.4 |
| Nothobranchius hassoni | 27.285556 | -9.477667 |
| Nothobranchius hassoni | 27.284444 | -9.4775 |
| Nothobranchius hassoni | 27.283333 | -9.466667 |
| Nothobranchius hassoni | 27.281389 | -9.199444 |
| Nothobranchius hassoni | 27.326667 | -9.144722 |
| Nothobranchius hassoni | 27.326389 | -9.14 |
| Nothobranchius interruptus | 39.783333 | -3.9 |
| Nothobranchius interruptus | 39.783333 | -3.9 |
| Nothobranchius interruptus | 39.603333 | -3.443056 |
| Nothobranchius interruptus | 39.304722 | -3.183333 |
| Nothobranchius interruptus | 39.766667 | -3.081667 |
| Nothobranchius interruptus | 39.766667 | -3.065 |
| Nothobranchius janpapi | 39.068889 | -8.451667 |
| Nothobranchius janpapi | 38.930833 | -8.038611 |
| Nothobranchius janpapi | 38.706389 | -7.9975 |
| Nothobranchius janpapi | 38.839167 | -7.906667 |
| Nothobranchius janpapi | 38.439444 | -7.825556 |
| Nothobranchius janpapi | 39.1375 | -7.816111 |
| Nothobranchius janpapi | 37.834444 | -7.721944 |
| Nothobranchius janpapi | 38.001667 | -7.718611 |
| Nothobranchius janpapi | 37.777778 | -7.419167 |
| Nothobranchius janpapi | 38.876944 | -7.099444 |
| Nothobranchius janpapi | 38.889444 | -6.7075 |
| Nothobranchius janpapi | 38.683333 | -6.683333 |
| Nothobranchius janpapi | 38.693889 | -6.678889 |
| Nothobranchius janpapi | 38.702778 | -6.654167 |
| Nothobranchius janpapi | 38.743333 | -6.635833 |
| Nothobranchius janpapi | 38.598333 | -6.610556 |
| Nothobranchius janpapi | 38.870833 | -6.490278 |
| Nothobranchius janpapi | 38.886111 | -6.462778 |
| Nothobranchius janpapi | 38.758611 | -6.037778 |
| Nothobranchius jubbi | 39.894167 | -3.199444 |
| Nothobranchius jubbi | 40.06 | -3.102778 |
| Nothobranchius jubbi | 40.133333 | -3.033333 |
| Nothobranchius jubbi | 40.133333 | -2.95 |
| Nothobranchius jubbi | 40.206111 | -2.670833 |
| Nothobranchius jubbi | 40.3325 | -2.439444 |
| Nothobranchius jubbi | 40.144444 | -2.348333 |
| Nothobranchius jubbi | 39.842778 | -2.291667 |
| Nothobranchius jubbi | 39.821944 | -2.049722 |
| Nothobranchius jubbi | 40.1 | -1.983333 |
| Nothobranchius jubbi | 40.116667 | -1.983333 |
| Nothobranchius jubbi | 40.083333 | -1.933333 |
| Nothobranchius jubbi | 40.255 | -1.900833 |
| Nothobranchius jubbi | 40.066667 | -1.9 |
| Nothobranchius jubbi | 40.001389 | -1.515556 |
| Nothobranchius jubbi | 39.856667 | -0.6925 |
| Nothobranchius jubbi | 42.603333 | 0.033333 |
| Nothobranchius jubbi | 41.836111 | 0.439722 |
| Nothobranchius jubbi | 41.672778 | 0.527778 |
| Nothobranchius jubbi | 41.627222 | 0.794167 |
| Nothobranchius jubbi | 41.976667 | 0.835833 |
| Nothobranchius jubbi | 43.248611 | 0.885 |
| Nothobranchius jubbi | 44.738611 | 1.941667 |
| Nothobranchius jubbi | 45.177778 | 2.3775 |
| Nothobranchius jubbi | 44.231667 | 2.407222 |
| Nothobranchius jubbi | 44.216667 | 2.9 |
| Nothobranchius jubbi | 44.095833 | 3.125833 |
| Nothobranchius jubbi | 44.733333 | 3.323611 |
| Nothobranchius jubbi | 45.208611 | 3.836111 |
| Nothobranchius kadleci | 34.1 | -20.683333 |
| Nothobranchius kafuensis | 24.028611 | -18.11 |
| Nothobranchius kafuensis | 24.365278 | -17.926389 |
| Nothobranchius kafuensis | 25.191111 | -17.752778 |
| Nothobranchius kafuensis | 26.164722 | -16.708056 |
| Nothobranchius kafuensis | 26.009722 | -16.529722 |
| Nothobranchius kafuensis | 27.655 | -16.035 |
| Nothobranchius kafuensis | 27.366667 | -15.966667 |
| Nothobranchius kafuensis | 28.574444 | -15.923333 |
| Nothobranchius kafuensis | 28.358611 | -15.786667 |
| Nothobranchius kafuensis | 25.9875 | -15.693611 |
| Nothobranchius kafuensis | 27.598611 | -15.664722 |
| Nothobranchius kafuensis | 26 | -15.05 |
| Nothobranchius kafuensis | 26.400833 | -15.049722 |
| Nothobranchius kafuensis | 25.254167 | -14.846944 |
| Nothobranchius kafuensis | 28.066667 | -14.533333 |
| Nothobranchius kilomberoensis | 36.961111 | -8.684167 |
| Nothobranchius kilomberoensis | 37.067222 | -8.524722 |
| Nothobranchius kilomberoensis | 37.170833 | -8.416944 |
| Nothobranchius kilomberoensis | 36.925 | -8.311111 |
| Nothobranchius kilomberoensis | 37.111944 | -8.281944 |
| Nothobranchius kilomberoensis | 36.7 | -8.166667 |
| Nothobranchius kirki | 35.5 | -15.666667 |
| Nothobranchius kirki | 35.532778 | -15.4 |
| Nothobranchius kirki | 35.533333 | -15.4 |
| Nothobranchius kirki | 35.532533 | -15.389183 |
| Nothobranchius kirki | 35.542222 | -15.329722 |
| Nothobranchius kirki | 35.582967 | -15.014133 |
| Nothobranchius kirki | 35.790833 | -14.863056 |
| Nothobranchius kirki | 35.816667 | -14.733333 |
| Nothobranchius kirki | 35.816944 | -14.732778 |
| Nothobranchius kiyawensis | 0.616667 | 5.766667 |
| Nothobranchius korthausae | 39.121111 | -7.980833 |
| Nothobranchius korthausae | 39.718611 | -7.955833 |
| Nothobranchius korthausae | 39.719983 | -7.94055 |
| Nothobranchius korthausae | 39.636944 | -7.933889 |
| Nothobranchius korthausae | 39.714167 | -7.9325 |
| Nothobranchius korthausae | 39.215 | -7.887222 |
| Nothobranchius korthausae | 39.730683 | -7.884467 |
| Nothobranchius korthausae | 39.740333 | -7.883133 |
| Nothobranchius korthausae | 39.741383 | -7.8816 |
| Nothobranchius korthausae | 39.794444 | -7.829444 |
| Nothobranchius korthausae | 39.794433 | -7.829417 |
| Nothobranchius korthausae | 39.819722 | -7.806944 |
| Nothobranchius korthausae | 39.840833 | -7.755 |
| Nothobranchius korthausae | 39.135556 | -7.205 |
| Nothobranchius korthausae | 39.124167 | -7.1975 |
| Nothobranchius korthausae | 39.387222 | -6.963611 |
| Nothobranchius korthausae | 39.248889 | -6.930556 |
| Nothobranchius krammeri | 40.324017 | -11.15675 |
| Nothobranchius krammeri | 40.316667 | -11.15 |
| Nothobranchius krysanovi | 36.85 | -17.65 |
| Nothobranchius kuhntae | 34.866667 | -19.833333 |
| Nothobranchius lucius | 36.6625 | -8.395833 |
| Nothobranchius lucius | 36.6805 | -8.276111 |
| Nothobranchius lucius | 36.698333 | -8.175 |
| Nothobranchius lucius | 36.6935 | -8.167778 |
| Nothobranchius lucius | 36.683333 | -8.166667 |
| Nothobranchius lucius | 36.415167 | -8.148 |
| Nothobranchius lucius | 39.8145 | -7.832778 |
| Nothobranchius lucius | 39.832833 | -7.803333 |
| Nothobranchius lucius | 39.75 | -7.75 |
| Nothobranchius lucius | 39.293889 | -7.3325 |
| Nothobranchius lucius | 39.124167 | -7.1975 |
| Nothobranchius lumumbashi | 27.4893 | -11.6667 |
| Nothobranchius makondorum | 38.424167 | -16.855 |
| Nothobranchius makondorum | 40.527833 | -13.394167 |
| Nothobranchius makondorum | 40.183333 | -12.5 |
| Nothobranchius makondorum | 40.1 | -11.866667 |
| Nothobranchius makondorum | 38.284722 | -10.928833 |
| Nothobranchius makondorum | 37.871944 | -10.924444 |
| Nothobranchius makondorum | 37.994722 | -10.910278 |
| Nothobranchius makondorum | 38.550556 | -10.8975 |
| Nothobranchius makondorum | 38.550556 | -10.8975 |
| Nothobranchius makondorum | 38.55 | -10.883333 |
| Nothobranchius makondorum | 37.746 | -10.877167 |
| Nothobranchius makondorum | 38.813333 | -10.551667 |
| Nothobranchius makondorum | 40.085 | -10.470278 |
| Nothobranchius makondorum | 38.4995 | -10.1605 |
| Nothobranchius malaissei | 29.900556 | -12.463611 |
| Nothobranchius malaissei | 29.428611 | -11.950278 |
| Nothobranchius malaissei | 29.076667 | -11.185556 |
| Nothobranchius malaissei | 28.753889 | -10.681667 |
| Nothobranchius malaissei | 28.288056 | -10.331667 |
| Nothobranchius malaissei | 28.016667 | -10.266667 |
| Nothobranchius malaissei | 28.133333 | -10.266667 |
| Nothobranchius melanospilus | 38.284167 | -10.928833 |
| Nothobranchius melanospilus | 37.869833 | -10.921333 |
| Nothobranchius melanospilus | 38.550333 | -10.8975 |
| Nothobranchius melanospilus | 39.098056 | -10.422778 |
| Nothobranchius melanospilus | 37.821389 | -9.965 |
| Nothobranchius melanospilus | 37.901667 | -9.904167 |
| Nothobranchius melanospilus | 37.581944 | -9.856667 |
| Nothobranchius melanospilus | 39.286667 | -9.805556 |
| Nothobranchius melanospilus | 37.481944 | -9.735833 |
| Nothobranchius melanospilus | 39.0275 | -9.589722 |
| Nothobranchius melanospilus | 39.632222 | -9.483056 |
| Nothobranchius melanospilus | 38.856667 | -9.315 |
| Nothobranchius melanospilus | 38.098611 | -9.305278 |
| Nothobranchius melanospilus | 38.994167 | -8.829444 |
| Nothobranchius melanospilus | 39.445556 | -8.824444 |
| Nothobranchius melanospilus | 38.826944 | -8.466667 |
| Nothobranchius melanospilus | 38.708333 | -8.401944 |
| Nothobranchius melanospilus | 39.016667 | -8.016667 |
| Nothobranchius melanospilus | 38.393333 | -8.015556 |
| Nothobranchius melanospilus | 38.854722 | -7.898611 |
| Nothobranchius melanospilus | 38.961667 | -7.865278 |
| Nothobranchius melanospilus | 39.745 | -7.842778 |
| Nothobranchius melanospilus | 39.832833 | -7.801933 |
| Nothobranchius melanospilus | 37.807222 | -7.778333 |
| Nothobranchius melanospilus | 38.81 | -7.640278 |
| Nothobranchius melanospilus | 37.539167 | -7.633333 |
| Nothobranchius melanospilus | 37.893611 | -7.625278 |
| Nothobranchius melanospilus | 36.75 | -7.538611 |
| Nothobranchius melanospilus | 37.716667 | -7.4 |
| Nothobranchius melanospilus | 38.206944 | -7.286389 |
| Nothobranchius melanospilus | 38.200556 | -7.282222 |
| Nothobranchius melanospilus | 39.086389 | -7.231389 |
| Nothobranchius melanospilus | 37.881389 | -7.213056 |
| Nothobranchius melanospilus | 39.135556 | -7.205 |
| Nothobranchius melanospilus | 39.124167 | -7.1975 |
| Nothobranchius melanospilus | 39.171944 | -7.187222 |
| Nothobranchius melanospilus | 37.933333 | -7.132222 |
| Nothobranchius melanospilus | 39.230833 | -7.101389 |
| Nothobranchius melanospilus | 38.618333 | -7.066944 |
| Nothobranchius melanospilus | 37.166389 | -7.041944 |
| Nothobranchius melanospilus | 37.752778 | -6.918611 |
| Nothobranchius melanospilus | 37.016667 | -6.816667 |
| Nothobranchius melanospilus | 37.403333 | -6.811389 |
| Nothobranchius melanospilus | 37.396944 | -6.795278 |
| Nothobranchius melanospilus | 37.199167 | -6.760278 |
| Nothobranchius melanospilus | 37.3225 | -6.758889 |
| Nothobranchius melanospilus | 37.335833 | -6.758611 |
| Nothobranchius melanospilus | 38.907222 | -6.730833 |
| Nothobranchius melanospilus | 37.120278 | -6.717778 |
| Nothobranchius melanospilus | 38.693889 | -6.678889 |
| Nothobranchius melanospilus | 39.125278 | -6.633889 |
| Nothobranchius melanospilus | 37.3225 | -6.520278 |
| Nothobranchius melanospilus | 38.870833 | -6.490278 |
| Nothobranchius melanospilus | 38.866667 | -6.483333 |
| Nothobranchius melanospilus | 38.886111 | -6.462778 |
| Nothobranchius melanospilus | 37.533889 | -6.4325 |
| Nothobranchius melanospilus | 37.266667 | -6.383333 |
| Nothobranchius melanospilus | 37.266667 | -6.383333 |
| Nothobranchius melanospilus | 38.77 | -6.373889 |
| Nothobranchius melanospilus | 37.886389 | -6.264444 |
| Nothobranchius melanospilus | 38.408056 | -6.241667 |
| Nothobranchius melanospilus | 37.22 | -6.235278 |
| Nothobranchius melanospilus | 36.731667 | -6.231389 |
| Nothobranchius melanospilus | 37.365556 | -6.154444 |
| Nothobranchius melanospilus | 37.455833 | -6.096389 |
| Nothobranchius melanospilus | 38.707222 | -6.095556 |
| Nothobranchius melanospilus | 39.240833 | -6.003611 |
| Nothobranchius melanospilus | 37.639722 | -5.896111 |
| Nothobranchius melanospilus | 37.395278 | -5.774444 |
| Nothobranchius melanospilus | 36.229167 | -5.539444 |
| Nothobranchius melanospilus | 38.951111 | -5.380556 |
| Nothobranchius melanospilus | 39.082222 | -5.176667 |
| Nothobranchius melanospilus | 39.056667 | -4.5625 |
| Nothobranchius melanospilus | 38.655278 | -4.529722 |
| Nothobranchius melanospilus | 39.3 | -4.516667 |
| Nothobranchius melanospilus | 39.253333 | -4.220556 |
| Nothobranchius microlepis | 38.978611 | -3.401389 |
| Nothobranchius microlepis | 40.083333 | -1.933333 |
| Nothobranchius microlepis | 40.066667 | -1.9 |
| Nothobranchius microlepis | 40.056389 | -1.7125 |
| Nothobranchius microlepis | 43.051111 | 2.1775 |
| Nothobranchius microlepis | 43.333333 | 2.333333 |
| Nothobranchius microlepis | 45.445833 | 3.898889 |
| Nothobranchius microlepis | 45.186667 | 3.917778 |
| Nothobranchius Mocimboa da Praia | 40.324017 | -11.15675 |
| Nothobranchius niassa | 37.6725 | -12.398611 |
| Nothobranchius niassa | 37.636667 | -12.191389 |
| Nothobranchius niassa | 37.534167 | -12.138056 |
| Nothobranchius niassa | 37.405 | -12.134722 |
| Nothobranchius niassa | 37.533333 | -12.133333 |
| Nothobranchius niassa | 37.436111 | -12.130278 |
| Nothobranchius niassa | 37.361389 | -12.129167 |
| Nothobranchius niassa | 37.560556 | -12.0875 |
| Nothobranchius Nyando River | 34.866667 | -0.216667 |
| Nothobranchius ocellatus | 38.166667 | -9.55 |
| Nothobranchius ocellatus | 37.903056 | -9.351944 |
| Nothobranchius ocellatus | 39.160833 | -8.131111 |
| Nothobranchius ocellatus | 38.708056 | -7.999167 |
| Nothobranchius ocellatus | 38.835833 | -7.908056 |
| Nothobranchius ocellatus | 38.416667 | -7.85 |
| Nothobranchius ocellatus | 37.75 | -7.675 |
| Nothobranchius ocellatus | 38.096111 | -7.651389 |
| Nothobranchius ocellatus | 38.965833 | -6.774444 |
| Nothobranchius ocellatus | 38.964167 | -6.688333 |
| Nothobranchius ocellatus | 38.770278 | -6.374167 |
| Nothobranchius oestergaardi | 29.833333 | -8.416667 |
| Nothobranchius orthonotus | 32.286667 | -27.550833 |
| Nothobranchius orthonotus | 32.266667 | -27.183333 |
| Nothobranchius orthonotus | 32.136944 | -27.091667 |
| Nothobranchius orthonotus | 32.2325 | -26.937222 |
| Nothobranchius orthonotus | 33.186944 | -24.561944 |
| Nothobranchius orthonotus | 32.009444 | -24.455833 |
| Nothobranchius orthonotus | 32.782222 | -24.411 |
| Nothobranchius orthonotus | 33.053056 | -24.3975 |
| Nothobranchius orthonotus | 32.975 | -24.35 |
| Nothobranchius orthonotus | 32.651667 | -24.299444 |
| Nothobranchius orthonotus | 31.981667 | -24.296111 |
| Nothobranchius orthonotus | 32.563 | -24.294 |
| Nothobranchius orthonotus | 31.935 | -24.244444 |
| Nothobranchius orthonotus | 31.931333 | -24.221389 |
| Nothobranchius orthonotus | 32.636389 | -24.22 |
| Nothobranchius orthonotus | 32.716667 | -24.05 |
| Nothobranchius orthonotus | 34.4 | -24.026111 |
| Nothobranchius orthonotus | 32.5671 | -23.474067 |
| Nothobranchius orthonotus | 31.943056 | -21.798056 |
| Nothobranchius orthonotus | 31.935278 | -21.772222 |
| Nothobranchius orthonotus | 34.208611 | -21.305833 |
| Nothobranchius orthonotus | 34.497222 | -20.696389 |
| Nothobranchius orthonotus | 34.102783 | -20.684267 |
| Nothobranchius orthonotus | 34.683056 | -19.826111 |
| Nothobranchius orthonotus | 34.935556 | -19.786389 |
| Nothobranchius orthonotus | 34.451389 | -19.399444 |
| Nothobranchius orthonotus | 34.9175 | -19.385833 |
| Nothobranchius orthonotus | 34.426944 | -19.225556 |
| Nothobranchius orthonotus | 34.533333 | -19.214444 |
| Nothobranchius orthonotus | 34.508611 | -19.17 |
| Nothobranchius orthonotus | 35.474722 | -18.656389 |
| Nothobranchius orthonotus | 36.715833 | -18.086111 |
| Nothobranchius orthonotus | 36.833333 | -17.75 |
| Nothobranchius orthonotus | 36.862233 | -17.661583 |
| Nothobranchius orthonotus | 35.302222 | -16.823056 |
| Nothobranchius orthonotus | 36.147222 | -16.599722 |
| Nothobranchius orthonotus | 34.770278 | -16.162778 |
| Nothobranchius orthonotus | 34.85 | -16.13 |
| Nothobranchius orthonotus | 35.25 | -15.07 |
| Nothobranchius orthonotus | 35.17 | -14.88 |
| Nothobranchius orthonotus | 34.73 | -14.47 |
| Nothobranchius orthonotus | 34.62 | -14.43 |
| Nothobranchius orthonotus | 34.77 | -14.43 |
| Nothobranchius orthonotus | 34.62 | -14.38 |
| Nothobranchius orthonotus | 34.643333 | -14.33 |
| Nothobranchius orthonotus | 34.793333 | -14.32 |
| Nothobranchius orthonotus | 34.543333 | -13.77 |
| Nothobranchius orthonotus | 34.37 | -13.72 |
| Nothobranchius orthonotus | 34.28 | -13.343333 |
| Nothobranchius orthonotus | 34.293333 | -13.21 |
| Nothobranchius palmqvisti | 38.949722 | -5.419444 |
| Nothobranchius palmqvisti | 39.099167 | -5.157222 |
| Nothobranchius palmqvisti | 38.915556 | -5.087222 |
| Nothobranchius palmqvisti | 39.01 | -4.574167 |
| Nothobranchius palmqvisti | 39.333333 | -4.533333 |
| Nothobranchius palmqvisti | 39.364167 | -4.505556 |
| Nothobranchius palmqvisti | 39.232778 | -4.190833 |
| Nothobranchius palmqvisti | 38.963333 | -4.076111 |
| Nothobranchius palmqvisti | 39.699722 | -3.954167 |
| Nothobranchius patrizii | 40.056389 | -2.908611 |
| Nothobranchius patrizii | 40.15 | -2.816667 |
| Nothobranchius patrizii | 40.171389 | -2.424722 |
| Nothobranchius patrizii | 40.3125 | -2.353611 |
| Nothobranchius patrizii | 39.920556 | -2.108889 |
| Nothobranchius patrizii | 40.126667 | -1.983333 |
| Nothobranchius patrizii | 42.355556 | 0.103333 |
| Nothobranchius patrizii | 42.643333 | 0.349722 |
| Nothobranchius patrizii | 42.916667 | 0.533333 |
| Nothobranchius patrizii | 42.920833 | 0.574167 |
| Nothobranchius patrizii | 42.082222 | 1.243889 |
| Nothobranchius patrizii | 44.447778 | 1.698611 |
| Nothobranchius patrizii | 43.091111 | 2.091667 |
| Nothobranchius patrizii | 44.950278 | 2.322222 |
| Nothobranchius patrizii | 43.628889 | 2.529167 |
| Nothobranchius patrizii | 45.544444 | 3.07 |
| Nothobranchius pienaari | 32.758333 | -24.491111 |
| Nothobranchius pienaari | 32.782222 | -24.411 |
| Nothobranchius pienaari | 32.766667 | -24.4 |
| Nothobranchius pienaari | 31.903611 | -24.3675 |
| Nothobranchius pienaari | 34.9275 | -24.337778 |
| Nothobranchius pienaari | 32.528333 | -24.3275 |
| Nothobranchius pienaari | 32.592778 | -24.3 |
| Nothobranchius pienaari | 32.6125 | -24.3 |
| Nothobranchius pienaari | 32.651667 | -24.299444 |
| Nothobranchius pienaari | 32.562778 | -24.295 |
| Nothobranchius pienaari | 32.563 | -24.294 |
| Nothobranchius pienaari | 34.878889 | -24.267222 |
| Nothobranchius pienaari | 31.931333 | -24.221389 |
| Nothobranchius pienaari | 34.703889 | -24.156111 |
| Nothobranchius pienaari | 32.642778 | -23.947778 |
| Nothobranchius pienaari | 32.661389 | -23.8175 |
| Nothobranchius pienaari | 35.229167 | -22.2875 |
| Nothobranchius pienaari | 35.043611 | -22.076944 |
| Nothobranchius pienaari | 34.102783 | -20.684267 |
| Nothobranchius rachovii | 34.866667 | -19.833333 |
| Nothobranchius rachovii | 34.620278 | -19.729167 |
| Nothobranchius rachovii | 34.949167 | -19.622222 |
| Nothobranchius rachovii | 34.526111 | -19.387222 |
| Nothobranchius rachovii | 35.341111 | -18.578333 |
| Nothobranchius rachovii | 35.854722 | -17.886111 |
| Nothobranchius rachovii | 36.862233 | -17.661583 |
| Nothobranchius rachovii | 36.857222 | -17.469444 |
| Nothobranchius robustus | 32.016667 | -2.766667 |
| Nothobranchius robustus | 32.343056 | -2.470556 |
| Nothobranchius robustus | 31.659444 | -0.851667 |
| Nothobranchius robustus | 31.266389 | -0.816111 |
| Nothobranchius robustus | 31.565556 | -0.658333 |
| Nothobranchius robustus | 31.6 | -0.55 |
| Nothobranchius robustus | 31.252222 | 0 |
| Nothobranchius robustus | 31.676944 | 0.0075 |
| Nothobranchius robustus | 32.281667 | 0.086111 |
| Nothobranchius robustus | 32.479722 | 0.115556 |
| Nothobranchius robustus | 32.5025 | 0.182778 |
| Nothobranchius robustus | 34.043056 | 0.214722 |
| Nothobranchius robustus | 31.595278 | 0.345833 |
| Nothobranchius robustus | 31.993611 | 0.352778 |
| Nothobranchius robustus | 33.19 | 0.444444 |
| Nothobranchius robustus | 30.925833 | 0.480833 |
| Nothobranchius robustus | 33.111944 | 0.554444 |
| Nothobranchius robustus | 31.984444 | 1.039167 |
| Nothobranchius robustus | 30.940278 | 1.220278 |
| Nothobranchius rubroreticulatus | 28.931667 | 10.504722 |
| Nothobranchius rubroreticulatus | 19.633333 | 10.833333 |
| Nothobranchius rubroreticulatus | 19.633333 | 10.866667 |
| Nothobranchius rubroreticulatus | 19.892222 | 10.936111 |
| Nothobranchius rubroreticulatus | 19.915 | 10.955556 |
| Nothobranchius rubroreticulatus | 32.766667 | 11.583333 |
| Nothobranchius rubroreticulatus | 15.15 | 11.966667 |
| Nothobranchius rubroreticulatus | 14.157222 | 13.03 |
| Nothobranchius rubroreticulatus | 13.764444 | 13.216944 |
| Nothobranchius ruudwildekampi | 39.171667 | -7.187222 |
| Nothobranchius ruudwildekampi | 39.166667 | -7.183333 |
| Nothobranchius ruudwildekampi | 39.231667 | -7.162222 |
| Nothobranchius seegersi | 33.68345 | -6.681167 |
| Nothobranchius seegersi | 33.751033 | -6.501533 |
| Nothobranchius seegersi | 32.801389 | -5.983611 |
| Nothobranchius seegersi | 32.8 | -5.983333 |
| Nothobranchius steinforti | 37.183333 | -6.766667 |
| Nothobranchius steinforti | 38.163333 | -6.339167 |
| Nothobranchius taeniopygus | 33.956667 | -6.775278 |
| Nothobranchius taeniopygus | 31.755556 | -6.681944 |
| Nothobranchius taeniopygus | 34.058611 | -6.595556 |
| Nothobranchius taeniopygus | 32.008889 | -6.461944 |
| Nothobranchius taeniopygus | 35.274167 | -6.056667 |
| Nothobranchius taeniopygus | 35.2625 | -6.035 |
| Nothobranchius taeniopygus | 35.168333 | -6.012778 |
| Nothobranchius taeniopygus | 33.964167 | -5.998056 |
| Nothobranchius taeniopygus | 35.098889 | -5.938889 |
| Nothobranchius taeniopygus | 35.250833 | -5.915278 |
| Nothobranchius taeniopygus | 35.055556 | -5.906111 |
| Nothobranchius taeniopygus | 34.066667 | -5.583333 |
| Nothobranchius taeniopygus | 33.900833 | -5.348889 |
| Nothobranchius taeniopygus | 35.706111 | -5.046111 |
| Nothobranchius taeniopygus | 35.845278 | -4.875278 |
| Nothobranchius taeniopygus | 33.104167 | -4.750556 |
| Nothobranchius taeniopygus | 35.845278 | -4.740556 |
| Nothobranchius taeniopygus | 32.935 | -4.100556 |
| Nothobranchius taeniopygus | 33.15 | -4.056111 |
| Nothobranchius taeniopygus | 32.265833 | -3.706667 |
| Nothobranchius taeniopygus | 33.252222 | -3.5975 |
| Nothobranchius taeniopygus | 33.266111 | -3.573056 |
| Nothobranchius taeniopygus | 31.890556 | -3.4125 |
| Nothobranchius taeniopygus | 33.281944 | -3.234167 |
| Nothobranchius taeniopygus | 32.469722 | -2.92 |
| Nothobranchius taeniopygus | 32.684444 | -2.732778 |
| Nothobranchius taeniopygus | 32.9 | -2.627222 |
| Nothobranchius taeniopygus | 33.475278 | -2.580556 |
| Nothobranchius taeniopygus | 34.115833 | 1.361111 |
| Nothobranchius thierryi | 0.45 | 10.383333 |
| Nothobranchius ugandensis | 32.278611 | 0.160556 |
| Nothobranchius ugandensis | 34.048889 | 0.281944 |
| Nothobranchius ugandensis | 32.305278 | 0.389722 |
| Nothobranchius ugandensis | 33.668889 | 0.469722 |
| Nothobranchius ugandensis | 33.516667 | 0.5 |
| Nothobranchius ugandensis | 33.120833 | 0.918889 |
| Nothobranchius ugandensis | 33.8675 | 0.939167 |
| Nothobranchius ugandensis | 33.033333 | 1.066667 |
| Nothobranchius ugandensis | 32.4125 | 1.261667 |
| Nothobranchius ugandensis | 31.386389 | 1.262778 |
| Nothobranchius ugandensis | 32.910556 | 1.307222 |
| Nothobranchius ugandensis | 33.798056 | 1.317778 |
| Nothobranchius ugandensis | 31.110278 | 1.523333 |
| Nothobranchius ugandensis | 33.770278 | 1.680556 |
| Nothobranchius ugandensis | 31.370833 | 1.749167 |
| Nothobranchius ugandensis | 33.075 | 2.046944 |
| Nothobranchius ugandensis | 32.47 | 2.093056 |
| Nothobranchius ugandensis | 33.997222 | 2.173611 |
| Nothobranchius ugandensis | 33.673333 | 2.198611 |
| Nothobranchius ugandensis | 33.606389 | 2.289167 |
| Nothobranchius ugandensis | 31.279167 | 2.394444 |
| Nothobranchius virgatus | 31.149167 | 6.886667 |
| Nothobranchius virgatus | 30.810278 | 7.185278 |
| Nothobranchius virgatus | 28.974167 | 10.484722 |
| Nothobranchius virgatus | 28.665 | 10.721667 |
| Nothobranchius virgatus | 28.082778 | 11.197222 |
| Nothobranchius virgatus | 28.383333 | 11.65 |
| Nothobranchius virgatus | 32.1 | 11.95 |
| Nothobranchius virgatus | 31.681111 | 12.801667 |
| Nothobranchius wattersi | 35.25 | -15.066667 |
| Nothobranchius wattersi | 35.169583 | -14.929717 |
| Nothobranchius wattersi | 34.733333 | -14.466667 |
| Nothobranchius wattersi | 34.616667 | -14.45 |
| Nothobranchius wattersi | 34.616667 | -14.433333 |
| Nothobranchius wattersi | 34.616667 | -14.433333 |
| Nothobranchius wattersi | 34.766667 | -14.433333 |
| Nothobranchius wattersi | 34.616667 | -14.383333 |
| Nothobranchius wattersi | 34.590917 | -14.347483 |
| Nothobranchius wattersi | 34.516667 | -14.166667 |
| Nothobranchius wattersi | 34.44735 | -13.851933 |
| Nothobranchius wattersi | 34.44735 | -13.851933 |
| Nothobranchius wattersi | 34.55 | -13.766667 |
| Nothobranchius wattersi | 34.283333 | -13.35 |
| Nothobranchius wattersi | 34.3 | -13.216667 |
